# Healthy apple program to support child care centers to alter nutrition and physical activity practices and improve child weight: a cluster randomized trial

**DOI:** 10.1186/s12889-017-4951-y

**Published:** 2017-12-19

**Authors:** Jodi D. Stookey, Jane Evans, Curtis Chan, Lisa Tao-Lew, Tito Arana, Susan Arthur

**Affiliations:** 1 San Francisco Department of Public Health, Maternal, Child & Adolescent Health, 30 Van Ness, Suite 260, San Francisco, CA 94102 USA; 2 Children’s Council of San Francisco, San Francisco, CA USA

**Keywords:** Child obesity, Weight change, Child care center

## Abstract

**Background:**

North Carolina Nutrition and Physical Activity Self-Assessment for Child Care (NAP SACC) resources improve child body mass index (BMI) when the resources are introduced by nurses to child care providers, and offered with workshops and incentives. In San Francisco, public health and child care agencies partnered to adapt NAP SACC resources into an annual “Healthy Apple” quality improvement program (HAP).

**Methods:**

This cluster randomized controlled trial pilot-tested integration of the HAP with bi-annual public health screenings by nurses. All child care centers that participated in Child Care Health Program (CCHP) screenings in San Francisco in 2011–2012 were offered routine services plus HAP in 2012–2013 (CCHP + HAP, *n* = 19) or routine services with delayed HAP in 2014–2015 (CCHP + HAP Delayed, *n* = 24). Intention-to-treat analyses (robust SE or mixed models) used 4 years of screening data from 12 to 17 CCHP + HAP and 17 to 20 CCHP + HAP Delayed centers, regarding 791 to 945 children ages 2 to 5y, annually. Year-specific, child level models tested if children in CCHP + HAP centers had greater relative odds of exposure to 3 index best practices and smaller Autumn-to-Spring changes in BMI percentile and z-score than children in CCHP + HAP Delayed centers, controlling for age, sex, and Autumn status. Multi-year, child care center level models tested if HAP support modified year-to-year changes (2013–2014 and 2014–2015 vs 2011–2012) in child care center annual mean Autumn-to-Spring BMI changes.

**Results:**

In 2011–2012, the CCHP + HAP and CCHP + HAP Delayed centers had similar index practices (<15% of children were exposed to a physical activity curriculum, staff joining in active play, and drinking water pitchers) and annual BMI changes. In 2013–2014: 60% of children in CCHP + HAP centers were exposed to the 3 index practices vs 19% in CCHP + HAP Delayed centers; Mean (SE) child BMI percentile (−2.6 (0.9), *p* = 0.003) and z-score (−0.08 (0.03), *p* = 0.007) decreased more in CCHP + HAP vs CCHP + HAP Delayed centers. In 2014–2015, after all centers were offered HAP, the index practices and BMI changes were improved for all centers vs 2011–2012.

**Conclusions:**

Integration of the HAP with existing public health nursing services was associated with significantly more children exposed to best practices and improvement in child BMI change. The results warrant continued integration of HAP into local public health infrastructure.

**Trial registration:**

ISRCTN18857356 (24/04/2015) Retrospectively registered.

**Electronic supplementary material:**

The online version of this article (doi:10.1186/s12889-017-4951-y) contains supplementary material, which is available to authorized users.

## Background

Childhood obesity prevention is a public health priority to reduce chronic disease risk [1]. In the United States, national initiatives, including the Caring for Our Children: National Health and Safety Performance Standards and the Let’s Move! Child Care campaign, call on child care providers to champion childhood obesity prevention efforts [2, 3]. Child care providers are in a unique position to educate parents and children about healthy eating and physical activity, and ensure a healthy environment for children for up to 35 h per week [4, 5]. Many children eat a majority of their meals in child care [6]. The national campaigns offer free, online resources for child care providers to learn about nutrition and physical activity best practices and engage in quality improvement [2, 3].

Nationally sponsored online resources for child care providers are based on work done by the University of North Carolina Nutrition and Physical Activity Self-Assessment for Child Care (NAP SACC) program. The NAP SACC program developed and tested resources which simultaneously promote a set of over 80 evidence-based best practices, and engage child care providers in an iterative quality improvement process [7–12].

Observational, quasi-experimental, and randomized controlled research studies describe effects of NAP SACC resources [7–12]. Implementation of NAP SACC resources is associated with significant improvement in child care provider practices, when the set of best practices is presented to child care providers as an iterative process involving self-assessment, choice of practices to improve, goal setting, action, and re-self-assessment [7–12]. Implementation of the NAP SACC resources and process are, furthermore, associated with significant improvement in child BMI, when the resources and process are introduced to child care providers by nurses, along with 5 h of educational workshops, and $500 participation incentive [12].

To bridge the gap between resources, which are endorsed by national sponsors and available online, and resources, which are effective when distributed by nurses, in-person, with tailored technical assistance, workshops, and financial incentives, the San Francisco Child Care Wellness Collaborative developed the Healthy Apple Program (HAP). The intent of the HAP was to provide local linkage of child care providers to resources and tailored support. With permission from NAP SACC, the HAP translated the nutrition and physical activity NAP SACC resources and process into a program that coordinates self-assessment and practice improvement across child care providers in San Francisco. The citywide coordination makes it possible to aggregate self-assessment data, identify common provider improvement goals and technical assistance needs, and tailor local response and workshops to efficiently address those goals and needs. The HAP added an award incentive for child care providers to adopt all NAP SACC best practices. Award schemes effectively incentivize child care providers to adopt nutrition best practices [13].

This paper reports the results of the HAP pilot evaluation. The evaluation aimed to determine if integration of HAP resources into routine public health nursing services significantly increased the number of nutrition and physical activity best practices adopted by child care centers, and improved changes in child BMI percentile and z-score, relative to routine public health nursing services. Prior to 2012–2013, public health nursing services offered to local child care providers by the San Francisco Department of Public Health Child Care Health Program (CCHP) did not systematically link child care providers to online resources, promote a comprehensive set of nutrition and physical activity best practices, or encourage providers to engage in iterative self-assessment and quality improvement. The HAP pilot evaluation essentially tested for effects of HAP resources on child BMI, given conditions similar to those specified by Alkon et al. [12] in the randomized trial of NAP SACC effects on child BMI. The evaluation contributes information about ways to implement NAP SACC resources in communities, to ultimately realize national child obesity prevention goals.

## Methods

### Cluster randomized controlled trial design

The HAP pilot evaluation was designed as a cluster randomized trial, with child care centers treated as clusters. The clustered design was selected because the CCHP serves child care providers, and does not have direct contact with the parents or guardians of children. The CCHP health workers and child care providers were not blinded to treatment allocation.

**Fig. 1:**
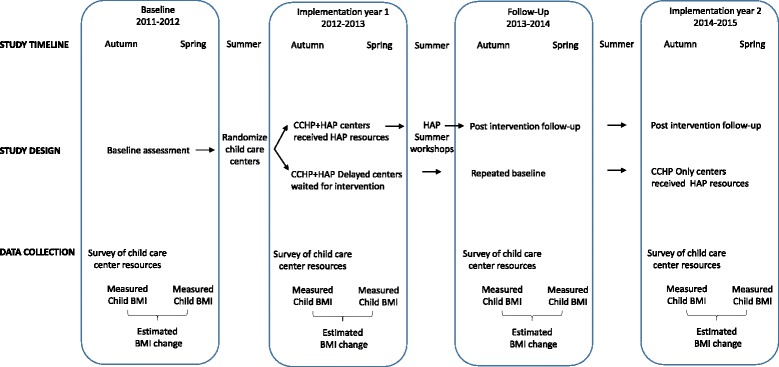
Timeline, study design, and data collection for the Healthy Apple Program pilot. BMI: Body mass index; CCHP: Child Care Health Program; HAP: Healthy Apple Program. In child care center level analysis, child care centers were followed, longitudinally, over multiple academic years. As the children attending the child care centers changed each year, in child-level analysis, each academic year was considered separately

Figure 1 provides an overview of the timeline, design and data collection for the HAP pilot evaluation. Child care centers were randomly allocated to receive routine CCHP services plus HAP (CCHP + HAP) or routine CCHP services plus HAP after a one-year delay (CCHP + HAP Delayed). HAP resources were offered to CCHP + HAP centers beginning in Spring 2013 through Summer 2013. To ensure that all child care centers received the best practice resources, child care centers allocated to the CCHP + HAP Delayed group were also offered HAP resources, only after a delay, in 2014–2015. The one-year delay allowed the evaluation to have a control group, as well as CCHP staff to stagger the outreach and technical assistance workload. Data were collected at the child level and child care center level, each year, according to the academic calendar, which begins with Autumn enrollment. The same measures were collected over time. There was no overlap between the Implementation year 1 activities and Follow-up year outcome measures in 2013–2014. The Follow-up year measures were collected in Autumn 2013, months after the Summer 2013 HAP workshops were completed.

The evaluation design allowed for comparison of the randomly allocated intervention and control groups in the Baseline year, in the Follow-up year (2013–2014, i.e. after the CCHP + HAP centers received HAP resources), as well as  in Implementation year 2 (2014–2015, i.e. after the CCHP + HAP Delayed centers received the HAP resources). The working hypothesis for this design was that the randomly allocated groups would differ significantly with respect to outcomes in the Follow-up year, but not in the Baseline year or Implementation year 2. The evaluation design also made it possible to describe within-group, pre-post intervention changes, e.g. changes between 2011 and 2012 and 2014–2015 for each group, as well as the whole cohort of child care centers served by CCHP.

### Target population

All child care centers that participated in CCHP nutrition screenings in 2011–2012 were eligible for the HAP pilot. The CCHP provides services to child care centers that primarily serve low income children in San Francisco and do not have federal, state or school district funding. All child care centers had the option to decline each voluntary CCHP screening and/or decline participation in HAP pilot activities. A $25 gift card was offered to one representative per child care center for participation in the HAP pilot. The target population inclusion criteria did not change after trial commencement. Child care centers that were eligible in 2011–2012 remained eligible for the duration of the pilot, even if they declined CCHP services in interim years.

The target population included all children, ages 2 to 5y, enrolled at eligible child care centers. Families consented to CCHP services, including use of data for public health program planning and evaluation, and could opt out of screenings at any time.

### Evaluation exclusion criteria

Child care centers that were closed in Autumn 2012 or declined CCHP services for 2012–2013 before the randomization were ineligible for the HAP pilot. Child care centers with funding from Head Start, the San Francisco Unified School District, or Community College District were ineligible to receive CCHP screenings, and excluded from the HAP pilot. Child care centers that declined one or both BMI screenings in any given year were excluded from evaluation analyses for that year, because of missing data regarding the primary outcome of interest, annual change in BMI between the Autumn and Spring screenings. Children who declined one or both screenings or were absent on the date(s) of screening in any given year were excluded from evaluation analyses for that year. No other exclusion criteria were applied.

### Randomization protocol

In Summer 2012, the SFDPH epidemiologist randomized child care centers in two blocks, one block for each of two CCHP health workers responsible for BMI screenings. Each health worker has a specific caseload. For each health worker, eligible child care centers were listed in alphabetical order. A list of the same length, of random, unique, unsorted numbers was generated using randomizer.org. For each health worker, child care centers had an equal chance of being assigned to CCHP + HAP or CCHP + HAP Delayed. Enrollment in the child care centers ranged from 14 to 160 children. The mean (SE) enrollment in child care centers did not vary significantly by treatment assignment (48 (9) vs 37 (4)), and remained stable over time.

### Allocated interventions

Table 1 summarizes the services and resources offered to the randomly allocated groups in Implementation year 1, 2012–2013. The HAP resources included an invitation packet, which included information about the HAP, a self-assessment for child care providers (see Additional file 1), and information about the gift card incentive for completing the self-assessment. The HAP resources also included a goal setting worksheet (see Additional file 2), hard copy Tip Sheets (see Additional file 3) and online Technical Assistance materials (http://healthyapple.arewehealthy.com/resources.aspx). After Implementation year 1, HAP resource distribution was paused until 2014–2015. In 2014–2015, all child care centers were invited to participate in the HAP.

**Table 1:** Intervention components for the HAP pilot evaluation

Abbreviation or acronym	Name of resource	Resource content
CCHP	Child Care Health Program	• Bi-annual BMI screenings offered by public health nurses or health workers at child care centers • Individual child health referral • Nutrition education (circle time for children)
HAP	Healthy Apple Program	• Free self-assessment materials, information about over 80 best practices, and technical assistance resources for child care providers • Citywide coordination of quality improvement processes for child care providers • Tailored workshops and award incentives for child care providers to adopt nutrition and physical activity best practices
CCHP + HAP	Child Care Health Program *Plus* Healthy Apple Program	• Bi-annual BMI screenings offered by public health nurses or health workers at child care centers • Individual child health referral • Nutrition education (circle time for children) • *Linkage of child care providers with HAP* • *Individualized technical assistance for providers* • Free self-assessment materials, information about over 80 best practices, and technical assistance resources for child care providers • Citywide coordination of quality improvement processes for child care providers • Tailored workshops and award incentives for child care providers to adopt nutrition and physical activity best practices

The Healthy Apple Program adapted resources developed by the University of North Carolina Nutrition and Physical Activity Self-Assessment for Child Care (NAP SACC) program. NAP SACC resources include information about over 80 evidence-based nutrition and physical activity best practices for child care providers serving preschool aged children. NAP SACC materials support providers to engage in an iterative quality improvement process, which involves self-assessment, choice of practices to improve, goal setting, action, and re-self-assessment

### Protocol for CCHP + HAP delayed centers

Throughout the evaluation period, routine CCHP services were given to centers allocated to the CCHP + HAP Delayed group. These services included public health nurse consultation, health education, and hearing, vision, dental, and nutrition screenings and referrals. Each academic year, in Autumn and Spring, the same two trained health workers visited all child care centers that accepted the free CCHP BMI screening. The protocol was essentially the same each year. Screening data were collected as described below (see measures section). Child-specific weight status reports were given to the child care centers to send home to the parents or caregivers. The current prevalence of overweight or obese children at the child care center was reported to the child care provider. In Autumn 2013, CCHP protocol also included drinking water promotion for all child care centers. The promotion included distribution of a pamphlet about the benefits of drinking water for child obesity prevention and child-sized water pitchers for all centers (Help-Yourself Pitchers, Lakeshore Learning, San Leandro, CA). In Implementation year 2, 2014–2015, CCHP + HAP Delayed centers were invited to participate in the HAP, and given HAP resources as described below.

### Protocol for CCHP + HAP centers

CCHP + HAP centers were offered the same services provided to CCHP + HAP Delayed centers. During Implementation year 1, in addition to the routine services, CCHP + HAP child care centers were invited to participate in the HAP pilot. CCHP public health nurses or health workers introduced the HAP resources and process, in-person, to child care center staff. They delivered the HAP invitation packet to the child care center, and spent up to 16 h per child care center, providing one-on-one support to each child care provider regarding the HAP self-assessment, goal setting, action plans to achieve the goal(s), Tip Sheets and online Technical Assistance resources. The CCHP staff had written instructions and form templates to standardize the delivery of one-on-one support (see Additional files 4 and 5).

In Summer 2013, the San Francisco Children’s Council offered two workshops to address needs identified by the HAP participants. A nutrition workshop addressed ideas for seasonal menu planning, child nutrition education resources for parents, and policies for food for holidays or celebrations. A physical activity workshop addressed how to integrate age-appropriate physical activity and academic learning for preschoolers (http://www.pkimbrell.com/). CCHP and HAP staff extended verbal and/or email invitations to the staff contact at each child care center for any of the center staff to attend the workshops. Child care centers were invited to participate, but not required, because all CCHP services are optional and voluntary. Child care center staff were not paid to attend the workshops.

In Autumn 2013, child care providers who completed the initial self-assessment were invited to re-take the self-assessment. Their most recent self-assessment scores were used to determine HAP award eligibility. A ceremony in Autumn 2013 recognized child care providers who met criteria for a HAP award (http://healthyapple.arewehealthy.com/HealthyAppleAward.aspx).

In Spring and Summer 2014, while the HAP pilot data and program structure were reviewed, CCHP staff did not offer HAP materials to any child care centers. HAP resources were made available to child care centers in the CCHP + HAP Delayed group, beginning in Autumn 2014.

### Data collection

CCHP health workers visited all child care centers that requested bi-annual BMI screenings in the Autumn and Spring of each academic year. The health workers recorded child age and sex, and measured child weight and height using a standardized protocol and calibrated instruments (SECA digital scale 874 and SECA Portable, standalone stadiometer with headboard, Hamburg, Germany). Measurements were taken after the child removed outer layers of clothing and shoes. The age- and sex-specific BMI percentile and BMI z-score for each child was calculated relative to the CDC 2000 growth reference [14] using Epi Info 7 software (CDC, Atlanta, GA). The change in BMI percentile was calculated. Incident cases of overweight or obesity were identified.

Every Autumn, the CCHP health workers recorded nutrition and physical activity resources that were visible to them during their visit. They interviewed the center staff about nutrition and physical activity resource needs. During the HAP evaluation period, in Autumn 2012, Autumn 2013, and Autumn 2014, the health workers gathered information about 3 practices which were relevant for tracking changes in response to HAP workshops that were offered in 2013: Use of a physical activity curriculum (yes/no); Staff usually join in physically active play with children (yes/no); and Pitchers of drinking water visible in the classroom (yes/no). Data regarding the 3 index practices were combined into a score to track and compare cumulative changes in these practices in all CCHP + HAP and CCHP + HAP Delayed centers. The score was a simple count of the number of index practices with possible values 0 to 3.

### Primary and secondary outcome measures

The primary outcome for the evaluation was annual Autumn to Spring change in child BMI percentile at the child level and child care center level. The annual change in BMI percentile was calculated as the difference between the Autumn and Spring screening results. The BMI change was also expressed as annual change in BMI z-score, given that z-scores can be compared across ages and can quantify extremes of the BMI distribution [15]. The incidence of overweight or obesity during the school year was measured as the proportion of children who were normal weight at the Autumn screening, who were overweight or obese at the Spring screening.

The secondary outcome was child’s relative odds of exposure to the 3 index practices available from data routinely collected by CCHP for all child care centers. At the child care center level, the index practice score was evaluated as a dichotomous variable (proportion of centers that had 2 or 3 index practices vs 0 or 1). At the child level, the score was expressed as a continuous variable, proportion of children exposed to 2 or 3 index practices.

### HAP process measures

HAP process measures, reflecting the intensity of exposure to HAP resources, were collected by the San Francisco Children’s Council Healthy Apple Program Coordinator. The process measures included the number of child care centers that completed the HAP self-assessment(s), set goals, received technical assistance materials, attended workshops, improved best practices, and received a HAP award. The proportion of children, who were potentially reached by child care provider participation in all steps of the HAP process from self-assessment to award recognition, was estimated.

### Statistical analyses

The HAP pilot evaluation used de-identified public health screening data, collected by the CCHP over four school-years (see Additional file 6). Statistical analyses were done using Stata/SE 9.2 software (StataCorp, College Station, TX). Missing data were assumed missing at random, because the primary reasons for missing data were shifts in funding for the child care centers, and provider preference to schedule only one screening visit each year. *P*-values less than 0.05 were considered statistically significant.

The evaluation population was described in terms of the flow of child care centers through the HAP pilot, age of children attending participating child care centers, and prevalence of child overweight or obesity within about 90 days after Autumn enrollment each year. The primary outcome, change in BMI percentile during the academic year, and related outcome measures including the change in BMI z-score and incidence of overweight or obesity during the year, were described by intervention group and year. The intracluster correlation coefficient (ICC), measure of within-child care center variance relative to between-child care center variance, was estimated to describe clustering in the outcome data in the Follow-up year and Implementation year 2.

### Intention-to-treat effects on the primary outcome

The CCHP + HAP vs. CCHP + HAP Delayed groups were compared in intention-to-treat analyses, which accounted for clustered data. Year-specific hierarchical linear models used child-level data to test for a main effect of time between the Autumn to Spring screenings during the academic year (Time) on child BMI percentile and z-score, and modification of the Time effect by invitation to the HAP (Time x HAP interaction). The year-specific models included child sex, age and overweight or obese status (yes/no) in the Autumn, and type of center (CCHP + HAP or CCHP + HAP Delayed) as predictors of the intercept, and CCHP + HAP vs CCHP + HAP Delayed status as predictor of the slope, i.e. annual change from Autumn to Spring. Year-specific model random-effect parameters used an independent covariance structure.

Year-specific logistic regression models used child level data to compare CCHP + HAP and CCHP + HAP Delayed child care centers with respect to the relative odds of incident overweight or obesity during the academic year. The logistic regression models adjusted for age, sex, and BMI percentile at the Autumn screening, and used robust standard errors to account for clusters of child care centers.

Multi-year hierarchical linear models combined child care center-level data from all 4 years to compare year-to-year change. These models tested if the child care center annual mean changes in BMI percentile and z-score in 2012–2013, 2013–2014 and 2014–2015 differed from the corresponding changes in 2011–2012 (main effects of Year), and if differences from year-to-year were modified by invitation to the HAP (significant Year x HAP interaction). The year-to-year models included the child care center mean child age, percent male, and prevalence of overweight or obesity in Autumn as predictors of the intercept, and HAP invitation as predictor of the slope. Year-to-year models specified an unstructured covariance structure.

### Pre-post intervention within-group change

The multi-year model provided information about pre-post intervention change over the four-year evaluation period for the CCHP + HAP and CCHP + HAP Delayed groups. The main effect of Year in the multi-year model represented an estimate of the change in outcome relative to the Baseline Year. If this change was not significantly modified by the Year x HAP interaction, then the estimate was interpreted as the estimated within-group change for both groups. Multi-year models without the Year x HAP interaction term were used to estimate the change in annual mean BMI changes between the Baseline Year and Implementation year 2 (2014–2015) for all centers served by CCHP.

### Intention-to-treat effects on the secondary outcome

The secondary outcome, proportion of children exposed to 2 or 3 index best practices, was described by intervention group and year. A year-specific logistic regression model, with robust standard errors, compared the CCHP + HAP and CCHP + HAP Delayed groups with respect to the relative odds of exposure to 2 or 3 best practices in 2013–2014.

### HAP process measures

The number of child care centers that were invited to the HAP, completed a self-assessment, set practice improvement goals, received technical assistance, attended workshops, improved practices, and received a HAP award was described by intervention group and year.

## Results

### Flow of child care centers through the HAP pilot

**Fig. 2:**
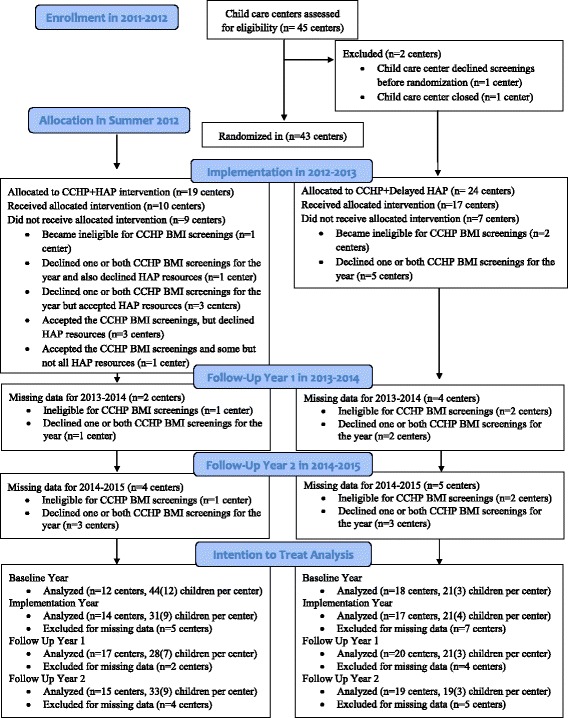
Flow of eligible child care centers through the Healthy Apple Program pilot. CONSORT: Consolidated Standards of Reporting Trials; CCHP: Child Care Health Program; HAP: Healthy Apple Program. Figure 2 was adapted from the CONSORT 2010 flow diagram [13]. Figure 2 describes the number of child care centers assessed for eligibility to participate, excluded from participation, randomized to CCHP + HAP or CCHP + HAP Delayed intervention groups, given allocated services and resources, followed in 2013–2014, and given resources in Implementation year 2 (2014–2015). The intention-to-treat analysis box reports the number of child care centers and mean (SE) number of children per child care center included in the analysis. The total number of children included in the analysis each year is reported in Table 2. It was not possible to estimate the cluster size of child care centers that were excluded from analyses, because enrollment data for non-participating centers were unavailable. After randomization, several child care centers became Head Start centers, which made them ineligible to receive CCHP services. All CCHP + HAP and CCHP + HAP Delayed centers were offered two free BMI screenings each year. The BMI screenings were offered every year, regardless of whether or not the child care center had previously declined BMI screening services. Child care centers that did not participate in both annual CCHP BMI screenings were missing data regarding annual BMI change and were excluded from analyses for that year. For this reason, the number of child care centers included in the analysis varied from year to year

A total of 43 child care centers were eligible to participate in the HAP pilot. Figure 2 describes the flow of eligible child care centers through the HAP pilot, according to the CONSORT 2010 guidelines [16]. The randomization allocated 19 child care centers to the CCHP + HAP group and 24 centers to the CCHP + HAP Delayed group. Child care centers that declined bi-annual BMI screening after randomization, in 2012–2013, 2013–2014, or 2014–2015, respectively, did not differ significantly from those that accepted screening with respect to the Autumn prevalence of overweight or obesity or likelihood of assignment to the CCHP + HAP or CCHP + HAP Delayed group in 2011–2012.

In 2012–2013, CCHP BMI screenings were not delivered as expected, because child care centers became ineligible for CCHP services due to unforeseen changes in Head Start funding, and/or child care centers declined one or both Autumn and Spring CCHP BMI screening(s) for the year. In the CCHP + HAP group, 5 centers did not participate in one or both CCHP BMI screenings in Autumn 2012 and/or Spring 2013. In the CCHP + HAP Delayed group, 7 centers declined one or both CCHP BMI screening(s) in 2012–2013.

Out of 14 CCHP + HAP centers that participated in both BMI screenings in 2012–2013, 11 centers completed the initial HAP self-assessment. Of these 11, 10 also received HAP technical assistance materials, and completed a HAP re-assessment in Fall 2013. Thus, 10 out of 19 CCHP + HAP centers received a full set of CCHP + HAP services plus resources, as allocated.

The number of child care centers and children included in the HAP evaluation analysis varied each year, depending on the availability of BMI change data (primary outcome). CCHP BMI screenings were offered to all eligible centers, every year, regardless of whether or not the centers had declined a BMI screening or HAP resources in prior years. Some child care centers that declined one or both BMI screening services in 2012–2013 accepted both BMI screenings in later years.

### Evaluation population characteristics

Table 2 describes the number of children who participated in both Autumn and Spring screenings each year. CCHP + HAP centers served significantly older children than CCHP + HAP Delayed centers in 2011–2012 and 2012–2013. Race-ethnic data were not available. Information about the average number of hours children spent in child care was not available. All CCHP + HAP centers and 22 out of 24 CCHP + HAP Delayed centers offered full-day programs.

**Table 2:** Number of child care centers and children that participated in bi-annual CCHP BMI screenings by year and group allocation

Year	Baseline		Implementation year 1		Follow-up		Implementation year 2	
	2011–2012		2012–2013		2013–2014		2014–2015	
Group Allocation	CCHP +HAP	CCHP +HAP Delayed	CCHP +HAP	CCHP +HAP Delayed	CCHP +HAP	CCHP +HAP Delayed	CCHP +HAP	CCHP +HAP Delayed
HAP Exposure	No	No	Yes	No	No	No	Yes	Yes
Number of child care centers randomized for the HAP pilot	19	24	19	24	19	24	19	24
Number of child care centers with complete BMI change data	12	18	14	17	17	20	15	19
Number of children enrolled in centers with complete data	522	380	430	361	529	416	502	353
Number of children by age								
2y	26	55	17	28	26	36	20	47
3y	207*	153	156	155	205	149	195	137
4y	282*	172	251*	175	293	223	251	154
5y	7	0	6	3	5	8	36	15

Study time was defined according to academic calendar year. *CCHP* Child Care Health Program, *BMI* Body mass index, *HAP* Healthy Apple Program; Child care centers were randomly allocated to the CCHP + HAP or CCHP + HAP Delayed group in Autumn 2012. Table 2 shows the total number of child care centers that were randomized, the number of centers that participated in both annual Autumn and Spring BMI screenings each year, and the number of children that participated in both BMI screenings each year, by intervention allocation. Complete data from both BMI screenings were required to calculate the primary outcome, annual change in BMI percentile between the Autumn and Spring screenings. Child care centers and children with missing BMI change data in any given year were excluded from the evaluation analyses for that year. *The age of children served differed significantly by intervention group assignment, *P* < 0.05

Table 3 describes the prevalence of overweight or obese children in the Autumn of each year. Over the 4-year HAP pilot period, the prevalence of overweight or obesity at Autumn enrollment increased significantly, by an estimated mean (SE) 4.3 (0.01) (*P* = 0.005) percentage points each year. The CCHP + HAP centers had a significantly smaller prevalence of overweight or obesity at Autumn enrollment, compared to CCHP + HAP Delayed centers, in the Baseline Year (2011–2012). In Autumn 2013 and Autumn 2014, CCHP + HAP and CCHP + HAP Delayed centers did not differ significantly with respect to the prevalence of overweight or obesity of children.

**Table 3:** Prevalence, incidence, and relative odds of overweight or obesity by year and group allocation

	Baseline			Implementation year 1			Follow-up			Implementation year 2		
Year	2011–2012			2012–2013			2013–2014			2014–2015		
Group allocation	CCHP +HAP (*n* = 522)	CCHP +HAP Delayed (*n* = 380)		CCHP +HAP (*n* = 430)	CCHP +HAP Delayed (*n* = 361)		CCHP +HAP (*n* = 529)	CCHP +HAP Delayed (*n* = 416)		CCHP +HAP (*n* = 502)	CCHP +HAP Delayed (*n* = 353)	
HAP exposure	No	No	.	Yes	No		No	No		Yes	Yes	
	%	%	Adj. OR (95%CI)	%	%	Adj. OR (95%CI)	%	%	Adj. OR (95%CI)	%	%	Adj. OR (95%CI)
Prevalence of overweight or obesity in the Autumn												
Age, y												
2–5	13	20	0.6 (0.4–0.9)*	22	33	0.6 (0.4–0.9)*	27	28	0.9 (0.6–1.5)	26	29	0.8 (0.5–1.2)
3	10	16	0.6 (0.3–1.3)	22	33	0.6 (0.4–0.9)*	28	28	1.0 (0.6–1.8)	27	32	0.8 (0.5–1.4)
4	17	26	0.6 (0.4–1.0)*	22	35	0.5 (0.3–0.9)*	27	30	0.8 (0.5–1.4)	29	32	0.9 (0.5–1.4)
Incidence of overweight or obesity between Autumn and Spring												
Age, y												
2–5	3.8	3.6	1.1 (0.5–2.3)	8.1	10.3	0.7 (0.3–1.5)	5.2	6.7	0.7 (0.4–1.2)	6.0	1.6	5.0 (1.2–1.4)*
3	3.2	3.9	0.8 (0.3–2.4)	5.0	9.6	0.4 (0.1–1.1)	6.8	6.5	1.2 (0.4–3.5)	7.0	1.1	9.1 (0.9–92.6)
4	4.3	3.1	1.4 (0.4–5.7)	10.2	8.8	1.2 (0.4–3.9)	4.2	7.1	0.4 (0.2–0.9)*	5.0	2.9	2.4 (0.4–12.7)

Study time was defined according to academic calendar year. *CCHP* Child Care Health Program, *HAP* Healthy Apple Program; Child care centers were randomly allocated to the CCHP + HAP or CCHP + HAP Delayed group in Autumn 2012; n: number of children; Adj. OR (95%CI): Adjusted odds ratio with 95% confidence interval; Overweight or obese was defined as age-and sex-specific BMI percentile ≥85%; Adjusted odds ratios were estimated using year-specific logistic regression models with robust standard errors, controlling for age, sex, and/or Autumn status; *Estimates for CCHP + HAP centers differed significantly from CCHP + HAP Delayed centers (reference), *P* < 0.05

### Intracluster correlation coefficients

In 2013–2014, the ICC for change in BMI percentile between Autumn 2013 and Spring 2014 was 0.04 (0.00–0.09); The ICC for incident overweight or obesity was 0.03 (0.00–0.08). In 2014–2015, the ICCs for change in BMI percentile and incident overweight or obesity were 0.05 (0.00–0.09) and 0.08 (0.01–0.15), respectively.

### Intention-to-treat effects on the primary outcome

Table 4 describes the unadjusted mean (SE) annual changes in BMI percentile and z-score for the CCHP + HAP and CCHP + HAP Delayed centers in each academic year. Table 3 describes the incidence of overweight or obesity by intervention group for each year. In the Baseline year, the CCHP + HAP and CCHP + HAP Delayed centers did not differ significantly with respect to the primary outcome, annual mean (SE) change in BMI percentile, BMI z-score, or incidence of overweight or obesity between the Autumn and Spring screenings.

**Table 4:** Mean (SE) changes in child BMI percentile and BMI z-score from Autumn to Spring by year and group allocation

Year	Baseline		Implementation year 1		Follow-up		Implementation year 2	
	2011–2012		2012–2013		2013–2014		2014–2015	
Group allocation	CCHP +HAP (*n* = 522)	CCHP +HAP Delayed (*n* = 380)	CCHP +HAP (*n* = 430)	CCHP +HAP Delayed (*n* = 361)	CCHP +HAP (*n* = 529)	CCHP +HAP Delayed (*n* = 416)	CCHP +HAP (*n* = 502)	CCHP +HAP Delayed (*n* = 353)
HAP exposure	No	No	Yes	No	No	No	Yes	Yes
Change in BMI percentile								
Age, y								
2–5	1.7 (0.6)	1.0 (0.7)	−2.2 (0.7)	−0.4 (0.7)	−2.7 (0.6)	−0.1 (0.6)	−0.7 (0.7)	−2.1 (0.7)
3	1.2 (1.0)	2.7 (1.2)	−2.5 (1.1)	0.9 (1.1)	−2.1 (1.0)	0.1 (0.9)	−0.3 (1.0)	−1.6 (1.2)
4	1.2 (0.8)	−0.7 (0.9)	−1.6 (0.9)	−2.1 (0.9)	−3.1 (0.8)	−0.9 (0.0)	−0.3 (1.0)	−1.1 (1.0)
Change in BMI z-score								
Age, y								
2–5	0.05 (0.02)	0 (0.02)	−0.1 (0.02)	−0.03 (0.03)	−0.1 (0.02)	−0.02 (0.02)	−0.04 (0.02)	−0.09 (0.02)
3	0.03 (0.03)	0.05 (0.04)	−0.1 (0.04)	0.01 (0.04)	−0.08 (0.04)	0 (0.03)	−0.04 (0.04)	−0.08 (0.05)
4	0.03 (0.03)	−0.06 (0.03)	−0.1 (0.03)	−0.1 (0.04)	−0.1 (0.03)	−0.05 (0.04)	−0.02 (0.04)	−0.06 (0.03)

Study time was defined according to academic calendar year. *CCHP* Child Care Health Program, *HAP* Healthy Apple Program; Child care centers were randomly allocated to the CCHP + HAP or CCHP + HAP Delayed group in Autumn 2012; n: number of children; BMI: Body mass index; Mean changes are unadjusted. All ages (2-5y) are included in the analysis. Age-specific estimates for age 2 and age 5 are not shown, due to small numbers (*n* < 5)

In the 2013–2014 year-specific models, the annual Autumn to Spring changes in BMI percentile and BMI z-score were significantly more negative in CCHP + HAP vs. CCHP + HAP Delayed centers (see Table 5). The incidence did not differ significantly by HAP participation for children ages 2–5 years or children age 3 years (see Table 3). The incidence of overweight or obesity was significantly lower, however, for children age 4 years in CCHP + HAP vs. CCHP + HAP Delayed centers.

**Table 5:** Estimated HAP effects on year-specific and year-to-year mean changes in BMI percentile and z-score

	Baseline		Implementation year 1		Follow-up		Implementation year 2	
Year	2011–2012		2012–2013		2013–2014		2014–2015	
HAP exposure for CCHP + HAP	No		Yes		No		Yes	
HAP exposure for CCHP + HAP Delayed	No		No		No		Yes	
Hierarchical linear model estimates	b (SE)	*P*	b (SE)	*P*	b (SE)	*P*	b (SE)	*P*
Year-specific model predicting child BMI percentile, %^a^								
Time	1.0 (0.7)	0.14	−0.4 (0.7)	0.57	−0.5 (0.7)	0.48	−2.7 (0.8)	0.001
Time x HAP	0.6 (0.9)	0.49	−1.7 (1.0)	0.08	−2.6 (0.9)	0.003	1.5 (1.0)	0.12
Year-specific model predicting child BMI z-score								
Time	0.00 (0.02)	0.88	−0.03 (0.03)	0.32	−0.03 (0.03)	0.25	−0.11 (0.03)	0.001
Time x HAP	0.04 (0.03)	0.18	−0.05 (0.04)	0.17	−0.08 (0.03)	0.007	0.06 (0.04)	0.11
Year-to-year model predicting child care center mean annual change in BMI percentile, %^b^								
Year	Reference		−0.2 (1.8)	0.90	0.01 (1.5)	0.99	−5.6 (2.2)	0.01
Year x HAP	Reference		−4.3 (2.7)	0.11	−5.1 (2.3)	0.03	−2.1 (2.4)	0.37
Year-to-year model predicting child care center mean annual change in BMI z-score								
Year	Reference		0.02 (0.06)	0.77	0.02 (0.05)	0.72	−0.2 (0.08)	0.02
Year x HAP	Reference		−0.2 (0.09)	0.06	−0.2 (0.08)	0.01	−0.1 (0.08)	0.18

*BMI* Body mass index, *BMI* percentiles and z-scores were calculated relative to the CDC 2000 growth reference; Study time was defined according to academic calendar year. *CCHP* Child Care Health Program, *HAP* Healthy Apple Program; Child care centers were randomly allocated to the CCHP + HAP or CCHP + HAP Delayed group in Autumn 2012; b (SE): model coefficient and standard error. P: p-value associated with the model coefficient

^a^Year-specific hierarchical linear models used child-level data to test for main effects of Time (change from Autumn to Spring during the school year) on BMI percentile and BMI z-score, as well as effect modification by invitation to the HAP (Time x HAP interaction). The year-specific models included child sex, age and overweight or obese status (yes/no) in the Autumn, and type of center (CCHP + HAP or CCHP + HAP Delayed) as predictors of the intercept and CCHP + HAP vs CCHP + HAP Delayed status as predictor of the slope, i.e. annual change from Autumn to Spring. Year-specific model random-effect parameters used an independent covariance structure

^b^Year-to-year hierarchical linear models used child care center-level data to test if the child care center annual mean changes in BMI percentile and z-score in 2012–2013, 2013–2014 and 2014–2015 differed from the corresponding changes in 2011–2012 (main effects of Year) and if differences from year to year were modified by invitation to the HAP (Year x HAP interaction). Year-to-year models included the child care center mean child age, percent male, and prevalence of overweight or obesity in Autumn as predictors of the intercept, and HAP invitation as predictor of the slope. Year-to-year models specified an unstructured covariance structure

In the 2014–2015 year-specific models, the annual changes in BMI percentile and z-score did not differ significantly by HAP assignment, consistent with delayed invitation of CCHP + HAP Delayed centers to the HAP. The incidence of overweight or obesity was significantly lower in CCHP + HAP Delayed vs CCHP + HAP centers (see Table 3).

In multi-year models that evaluated year-to-year changes at the child care center level, allocation to the CCHP + HAP group significantly modified change in the annual mean changes in BMI percentile and BMI z-score from the Baseline year to Follow-up year (see Table 5). Allocation to the CCHP + HAP group did not significantly modify the corresponding difference between the Baseline year and Implementation year 2, consistent with the delayed invitation of CCHP + HAP Delayed centers to the HAP.

### Pre-post intervention within-group change

In Implementation year 2, the BMI percentile and z-score decreased significantly in both the CCHP + HAP and CCHP + HAP Delayed groups, between Autumn 2014 and Spring 2015 (significant main effects of time, with no significant time x HAP interaction in the year-specific model) (see Table 5). In 2014–2015, the annual changes in BMI percentile and z-score in the CCHP + HAP Delayed group were more negative than they were in 2012–2013 or 2013–2014 (see Table 4). The annual changes in BMI percentile and z-score in the CCHP + HAP Delayed group in 2014–2015 were of similar magnitude to the changes in BMI percentile and z-score observed in 2013–2014 in the CCHP + HAP centers (> −0.2 and −0.1, respectively). The statistically significant main effect of Year with no significant Year x HAP interaction for Implementation year 2 in the multi-year model indicated a significant decrease in child BMI outcomes for both CCHP + HAP and CCHP + HAP Delayed groups relative to the Baseline year.

Before HAP implementation, the incidence of overweight or obesity among 4-year old children in the CCHP + HAP Delayed group, changed in parallel with that in the CCHP + HAP group. In the Follow-up year, the incidence among 4-year old children in CCHP + HAP centers decreased, but remained elevated in the CCHP + HAP Delayed centers, such that the incidence differed significantly between groups. In Implementation year 2, the difference between groups disappeared as the incidence in CCHP + HAP Delayed centers decreased (see Table 3).

For all centers combined, the estimated mean (SE) Autumn to Spring change in BMI percentile was −5.6 (2.5) points lower (*P* = 0.03) in 2014–2015 compared to the corresponding change in 2011–2012. The mean (SE) change in BMI z-score was −0.2 (0.09) lower (*P* = 0.02) in 2014–2015 vs. 2011–2012.

### Intention-to-treat effects on the secondary outcome

In Autumn 2012, before HAP implementation, the CCHP + HAP and CCHP + HAP Delayed centers did not differ significantly with respect to the proportion of children exposed to a physical activity curriculum, staff joining in active play, and/or pitchers of drinking water in the classroom. In Autumn 2012, 9% of children in the CCHP + HAP centers and 12% of children in CCHP + HAP Delayed centers were exposed to 2 or 3 of these best practices.

In Autumn 2013, the proportion of children exposed to 2 or 3 best practices was 60% in CCHP + HAP centers vs 19% of children screened in CCHP + HAP Delayed centers. Children enrolled in CCHP + HAP centers were over 6 times more likely to experience staff joining in active play, a physical activity curriculum, and/or have a pitcher of drinking water in the classroom than children in CCHP + HAP Delayed centers (OR: 6.5, 95%CI: 1.1–40.6). In child care centers that received the HAP invitation materials, but declined to complete the self-assessment or participate in activities, the proportion of children exposed to 2 or 3 best practices increased to 65%.

In Autumn 2014, 60% of children in CCHP + HAP centers and 62% of CCHP + HAP Delayed centers were exposed to 2 or 3 of the index best practices. In CCHP + HAP centers that actively participated in HAP, 85% of children were exposed to 2 or 3 of the index best practices.

### HAP process measures

Table 6 describes child care center participation in the HAP pilot. All centers that were randomly assigned to the CCHP + HAP group in 2012–2013 received a copy of the HAP self-assessment with invitation to participate in the HAP pilot. Between Spring 2012 and Autumn 2013, 10 CCHP + HAP centers made a total of 210 practice and/or policy improvements. The number of improvements per child care center ranged from 5 to 36. Almost two thirds of the children in child care centers assigned to CCHP + HAP were exposed to the HAP process from self-assessment to re-assessment.

**Table 6:** Number of child care centers and children involved in the HAP pilot in San Francisco

	CCHP + HAP centers were invited to HAP in 2012–2013			CCHP + HAP Delayed centers were invited to HAP in 2014–2015		
HAP process measures	Number of child care centers	Number of children enrolled	% of children enrolled	Number of child care centers	Number of children enrolled	% of children enrolled
Number randomized	19	–	–	24	–	–
Number with complete BMI change data	14	430	100	19	353	100
Number with complete BMI change data & completed HAP self-assessment	10	259	60	3	38	11
Set at least one best practice goal	9	231	54	1	22	6
Number of goals set						
0	4	342	40	0	0	0
1–3	4	140	16	0	0	0
4–6	6	378	44	0	0	0
Received technical assistance materials	9	231	54	3	38	11
Attended nutrition workshop	2	60	14	0	0	0
Attended physical activity workshop	3	76	18	0	0	0
Completed second self-assessment	10	259	60	–	–	–
Improved best practices	7	139	32	–	–	–
Received a HAP award	9	247	57	0	0	0

Child Care Health Program (CCHP); Healthy Apple Program (HAP); BMI: Body mass index

## Discussion

In the HAP pilot evaluation, integration of the HAP into existing public health infrastructure was associated with significant increases in the proportion of children exposed to nutrition and physical activity best practices, and significant reduction in child BMI and incident overweight or obesity. Implications of the results and potential for causal inference  are discussed in relation to aspects of the evaluation design.

Effects of public health interventions are complex to interpret, given variation in setting or contextual factors, multiple levels of intervention, and interacting components [17]. Many potentially confounding and/or effect modifying factors, including local, Statewide, and National initiatives, were operating in the background during the HAP pilot evaluation period. The HAP pilot intervened at the child care center level, but measured outcomes at the child level with no control for individual child level behavioral risk factors. The HAP had ample opportunity for interacting components, given that over 80 best practices were promoted, simultaneously, and offered with BMI screening and technical assistance by public health professionals.

### Contextual factors

Multiple factors in the background setting or context for the HAP pilot may confound or explain the HAP pilot outcomes. Between 2011 and 2015, several initiatives were concurrent with the HAP pilot in San Francisco. Pre-School-for-All subsidies were distributed to child care centers to offer physical activity classes. A citywide Rethink Your Drink media campaign was launched. The CCHP distributed child-sized pitchers of drinking water to child care providers to encourage children to drink water. Statewide, between 2010 and 2014, the prevalence of obesity among children ages 2 to 5 years enrolled in WIC decreased by −1.7 (−1.8, −1.6) percentage points from 18.4% to 16.6% [18]. California was one of 34 out of 56 States, nationwide, to observe a significant decrease in the prevalence of obesity among WIC enrolled children ages 2 to 5 years during the HAP pilot evaluation period [19]. Statewide and Federal efforts, such as the USDA’s revision of the WIC food package, CDC’s Early Care and Education Childhood Obesity program, and Let’s Move! Initiative, could have contributed to decreases in child obesity during the HAP evaluation period [18, 19]. To limit confounding effects of concurrent initiatives, correlated factors and/or trends, the HAP pilot evaluation used a randomized controlled design. It is unknown, nevertheless, if or what background factors were key conditions for outcomes of the HAP pilot.

Consistent with Statewide and National trends [18, 19], the annual Autumn to Spring changes in child BMI percentile, BMI z-score, and incidence of overweight or obesity in children age 4 years were significantly more negative in both the intervention (CCHP + HAP) and control (CCHP + HAP Delayed) groups in 2013–2014, relative to baseline. The significantly steeper decrease in annual BMI change in the CCHP + HAP group vs CCHP + HAP Delayed in the Follow-up year, and the disappearance of this difference in Implementation year 2, after the CCHP + HAP Delayed group also received HAP resources, suggest a ‘true’ beneficial effect of the local HAP beyond background trends.

### Intervention at the child care center level

Results from one other randomized controlled trial provide support for a ‘true’ HAP effect. Alkon et al. [12] report that implementation of NAP SACC resources at the child care center level result in a significantly more negative mean change in child BMI z-score over 7 months [12]. For children ages 2–5 years, who are becoming more mobile, decreases in BMI are normal; Increases in BMI during this period are considered a red flag for possible risk of overweight [20].

The HAP pilot evaluation and trial by Alkon et al. [12] shared several features in common, beyond implementation of NAP SACC resources at the child care center level. Both trials were restricted to child care centers that serve low income children, who are at higher risk of overweight or obesity than higher income children [21, 22]. Both trials involved time spent by nurses working one-on-one with providers, paid incentives for participation, and workshops about structured physical activity. The HAP pilot paid $25 to child care providers who completed the initial self-assessment. The Alkon et al. [12] intervention paid participants $500 to purchase equipment or supplies for physical activity. Both analyses followed children for BMI change for about 6 months. It is unknown if any or all of the similarities between trials, the social and behavioral risk factor profile of the population served, background promotion of physical activity and drinking water, nurse contact, in-person technical assistance, paid incentives, and/or length of follow-up were key conditions for the observed effects.

Replication of the HAP or NAP SACC effects may depend on specifying conditions similar to those specified by the present evaluation and trial by Alkon et al. [12]. Recent systematic reviews report that current evidence for effective strategies to implement nutrition and physical activity best practices in child care and improve child weight is weak and inconsistent [23]. Between-intervention differences, such as differences in how resources are distributed (online vs in-person by a trusted health professional with technical assistance), may explain inconsistent effects in the literature [23].

Unlike the HAP pilot, which invited all child care centers eligible for CCHP services in one county, the Alkon et al. [12] trial involved child care centers selected from 3 communities in different states, based on racial diversity, staff language, and kitchen availability. Whereas the HAP pilot included data for all children who were screened in the Autumn and Spring, the Alkon et al. [12] trial restricted the child-level analysis to children without chronic illness, conditions that affect nutritional status, severe food allergies, GI disorders, or mobility impairment. The HAP evaluation involved a greater number of child care centers and children. The substantive content of the interventions differed. The Alkon et al. [12] trial addressed the type of milk served, healthy snacks, written nutrition and physical activity policies, and information sheets for parents. Significant BMI change effects in both studies, despite these differences, suggest that the intervention effectiveness is not limited to select communities, child care centers, children or a specific mix of workshops.

Significant effects of intervention at the child care center level on BMI measures, which were collected at the child level, suggest some homogeneity of experience during the school day for children enrolled in the child care centers. Neither the HAP evaluation nor the trial by Alkon et al. [12] controlled for individual child behavioral risk factors over time.

Out of 43 child care centers randomized, 41 centers offered full-day programs. In Autumn 2012, over 90% of the child care centers served by CCHP provided Child and Adult Care Food Program (CACFP) funded meals (CCHP Program data), which have standardized composition for all children, daily, all year long. The intraclass correlation coefficients (ICC) estimated in the HAP pilot indicate greater variance between- vs within-child care centers than is typical in human studies, where the ICCs are usually between 0.01 and 0.02 [24]. If between-center variation in risk factors drives BMI change and incident overweight or obesity in child care centers, then HAP implementation at the child care center level may be relevant and effective for obesity prevention in child care centers.

### Interacting components

The HAP pilot linked child care center staff to resources that promote over 80 nutrition and physical activity best practices. The multifactorial approach recognizes obesity as the product of multiple interacting determinants. It provides opportunity for interactive, synergistic efforts, as well as incremental improvements, consistent with child care provider readiness and resources. Implementation of NAP SACC resources is associated with improvements in child care provider practices in several communities, in Arizona, California, Connecticut, North Carolina, Maine, and South Dakota [7–12, 25–27]. In the HAP pilot, each of the child care centers allocated to the CCHP + HAP group improved at least 5 practices and/or policies.

Child care centers varied with respect to completion of the HAP self-assessment(s), number of goals set, receipt of technical assistance materials, workshop attendance, and HAP award recognition. Two results suggest that the observed HAP effects were not contingent on exposure to all HAP resources. Child care centers which formally declined to participate in HAP, whose only HAP exposure was receiving the HAP invitation materials, made improvements consistent with HAP. Furthermore, the weight change advantage of CCHP + HAP centers that was observed in 2013–2014 disappeared in 2014–2015, after the CCHP + HAP Delayed centers simply received HAP invitation materials from CCHP staff. Only 3 out of 24 CCHP + HAP Delayed centers returned a completed HAP self-assessment in 2014–2015. None attended a workshop before the Spring 2015 BMI screenings were completed. The results suggest that systematic linkage of child care providers to HAP resources by CCHP staff, by itself, may have be effective for significantly reducing annual child BMI change. It remains to be determined if/how continued and/or greater intensity exposure to HAP resources might magnify benefits on child BMI and obesity prevention.

The HAP added to NAP SACC resources by coordinating self-assessment and improvement processes across providers, and by offering local award recognition as an incentive for practice improvement. Monitoring and aggregating HAP self-assessment data from providers, citywide, makes it possible for the HAP to tailor technical assistance and workshops to local needs and goals. The annual local award recognition raises awareness about nutrition and physical activity standards among child care providers, child care funders, parents, and pediatricians.

The HAP pilot delivered the HAP resources in combination with bi-annual child BMI screening by public health nurses and health workers. It is unlikely, that observed HAP effects were due to child care provider awareness of their center’s overall BMI change results for the previous year. Although annual information about the child care center’s mean Autumn to Spring change in child BMI percentile and z-score gives each child care provider a way to gauge if their efforts to improve nutrition and physical activity practices beneficially impact the children they serve during the academic year, each child care center’s mean child BMI change during the baseline year (2011–2012) was not systematically discussed with child care providers as part of the HAP implementation in 2012–2013. Before 2016–2017, CCHP only reported child- and timepoint-specific results to the child care providers. It remains to be determined if/how annual feedback about child BMI change can enhance the self-assessment process developed by NAP SACC.

### Strengths & Limitations

Strengths of the HAP pilot evaluation include generalizability of the evaluation population to all children served by child care centers with Preschool-For-All funding in San Francisco. The pilot covered all children enrolled in participating centers, as opposed to a sampled population. The primary outcome, child BMI change, was measured in a standardized way using calibrated instruments, over 4 years, by the same two health workers. Unlike obesity prevalence at Autumn enrollment, which is informative about the past experience of children before they entered care, child BMI change is a sensitive index of conditions when the children are in care, during the academic year.

Integration of the HAP with CCHP was a low-cost intervention. The HAP operation required 1FTE staff person at the local child care agency to coordinate self- assessment and quality improvement processes across child care centers, collect HAP process data, and organize workshops. HAP operation costs were covered through grant funds of less than $100,000 per year. The integration of HAP with CCHP required CCHP staff to spend approximately 16 h more per child care center than routine services. CCHP staff time for the 4-year HAP pilot, including time for public health nurse outreach and measured BMI change on almost 1000 children, annually, was provided in-kind by the public health department.

Each year, bi-annual BMI screening occurs systematically in child care centers across the United States. Head Start child care centers, for example, are mandated to screen child BMI at Autumn enrollment and again in the Spring. If addition of HAP support to health screening services significantly improves child BMI in San Francisco, similar linkage of child care providers to NAP SACC resources through child care health screening programs might be possible, inexpensive, and effective, nationwide.

The HAP pilot had several limitations. Randomization may not have eliminated bias related to treatment assignment, due to the relatively small number of child care centers and uneven demographic distribution across child care centers. Randomization would not have controlled for time-varying factors that happened to correlate with HAP exposure, and independently predict changes in child BMI. The health workers who collected the outcome data were not blinded to the intervention allocation. The income, race-ethnicity, and social determinants of health of the children were unknown, and not controlled in the design or analysis. Information was not available about child care centers and children that chose not to participate in both annual BMI screenings.

Potential for measurement bias also limits interpretation of the results in several ways. CCHP routine services did not include tracking of all NAP SACC components, in all child care centers, over time. CCHP providers reported difficulty understanding some of the HAP self-assessment questions. The best practice measures used in this evaluation were subject to variation in provider interpretation. Due to lack of unique child identifiers across child care centers, the analysis did not track the BMI changes of the same children across years. Child care providers reported barriers to improvement that were beyond their control, such as neighborhood crime, which were beyond the scope of the HAP to solve. This study tested a combined exposure, the HAP combined with linkage of child care providers to the HAP by public health staff. It did not evaluate the effect of the HAP alone.

## Conclusions

In this cluster randomized trial, the addition of HAP support to routine CCHP nursing services was associated with significantly greater improvements in child care provider practices and child BMI changes than routine CCHP services alone. The difference in outcomes was observed despite availability of free online resources [2, 3], throughout the study period, and concurrent local, Statewide, and national diet and activity initiatives [18, 19]. The results suggest that NAP SACC research, which was conducted with controlled experimental conditions and selected study population, may be effectively translated into public health programs to decrease the prevalence of childhood obesity. The results warrant continued integration of the HAP into public health and early childhood education infrastructure in San Francisco. The results suggest opportunity for child care health screening policy and protocol to systematically link child care providers to resources for practice improvement and child obesity prevention, nationwide.

## Additional files

Additional file 1: Healthy Apple Award Self-Assessment, Excellence in Nutrition, Physical Activity & Screen Time in Child Care Environments. CCHP health workers gave this document in hard-copy paper format to child care providers for self-assessment in 2013. (PDF 636 kb)

Additional file 2: Healthy Apple Award, Excellence in Nutrition, Physical Activity, & Screen Time in Child Care Environments, Goal Setting Worksheet. The CCHP health workers used this document to support child care providers to set goals and develop an action plan for practice improvement. (PDF 119 kb)

Additional file 3: Healthy Apple Award, Excellence in Nutrition, Physical Activity, & Screen Time in Child Care Environments, Technical Assistance Tip Sheets. Description: The CCHP health workers used and offered this document as a resource for child care providers to plan practice improvements. (PDF 25251 kb)

Additional file 4: Title: Healthy Apple Award Consultant Follow Up Instructions, June 4, 2013. Description: CCHP health workers followed the instructions detailed in this document to provide one-on-one support to child care providers after they completed the initial HAP self-assessment. (PDF 65 kb)

Additional file 5: Example Healthy Apple Program Consultant Guide. Description: The CCHP health workers used this template to summarize the target best practices and action plans that child care providers selected. (PDF 454 kb)

Additional file 6: HAP Pilot de-identified analysis file. This de-identified dataset includes the variables used for the reported analyses. The variables included were: Age- and sex-specific BMI percentile; BMI z-score; Intention-to-treat group assignment (springitts); Age in years; Sex (male = 1; female = 0), Dichotomous indicator of weight status at the Autumn screening (1 = BMI > = 85th percentile, 0: BMI < 85th percentile); and unique individual identifier (analysis generated id). (TXT 46 kb)

## Abbreviations

BMI: Body mass index
CCHP: Child Care Health Program
CONSORT: Consolidated Standards of Reporting Trials
HAP: Healthy Apple Program
ICC: Intracluster correlation coefficient
NAP SACC: University of North Carolina Nutrition and Physical Activity Self-Assessment for Child Care
SFDPH: San Francisco Department of Public Health
