# Supplementary material for: Healthy apple program to support child care centers to alter nutrition and physical activity practices and improve child weight: a cluster randomized trial
Source: BMC Public Health. 2017 Dec 19;17:965. doi: 10.1186/s12889-017-4951-y (PMC6389251; doi:10.1186/s12889-017-4951-y)
Supplement: Supplementary file 1 — Healthy Apple Award Self-Assessment, Excellence in Nutrition, Physical Activity & Screen Time in Child Care Environments. CCHP health workers gave this document in hard-copy paper format to child care providers for self-assessment in 2013. (PDF 636 kb) [file 12889_2017_4951_MOESM1_ESM.pdf]

Healthy Apple Award Self-Assessment

Excellence in Nutrition, Physical Activity, & Screen Time in Child Care Environments

Provider Name: ______________________________

Facility Name: ______________________________

Type of Facility: ☐ Family Child Care Home ☐ Center

Number of Children Enrolled: _____________

For more information, visit our website at
https://sites.google.com/site/childcarewellnesscollaborative/
(415) 355-6287 • healthyappleaward@gmail.com

The following organizations endorse the best practices of nutrition, physical activity, and screen time included in the Healthy Apple Award Self-Assessment:

Healthy Apple Award

Excellence in Nutrition, Physical Activity, & Screen Time in Child Care Environments

Dear Child Care Provider,

Thank you for taking the time to participate in the San Francisco Healthy Apple Award program. This should take no more than 30 minutes to complete. We hope that completing this self-assessment of your program's practices in the areas of nutrition, physical activity and screen time will help empower you to create the best environment for nurturing and encouraging healthy habits in the young children that you serve. Additionally, it will assist the Child Care Wellness Collaborative in recognizing you for your successes in these areas and supporting any goals for improvement you might have.

We expect that you will discover that you already incorporate many of these practices in your child care. We have designed an annual award system to recognize those of you who are meeting or exceeding the recommended best practice standards. Taking this self-assessment is just the beginning of the support system that we are offering.

You will receive a small incentive upon completion of your self-assessment (while supplies last). A short time after completing the self-assessment, a staff member will review it with you and determine your award level. You will work with this staff member to determine areas for improvement and set one or two achievable goals. The staff member will provide technical assistance and resources throughout the year to support you in achieving your goals. In addition, we will offer periodic workshops aimed at helping you to improve in each of the key areas. You are welcome to retake the self-assessment multiple times during the year in order to improve your award level, but incentives will only be available once a year. At this time, we plan to recognize award recipients in the fall.

The program and the self-assessment were adapted from the Nutrition and Physical Activity Self-Assessment for Child Care (NAP SACC) and the Let's Move Child Care Checklist. We hope that you will find it to be straightforward and helpful in highlighting areas of excellence and areas where extra support is needed. Please visit our website at https://sites.google.com/site/childcarewellnesscollaborative/ to learn more and find valuable resources.

As a voluntary program, participation is in NO WAY tied to funding or licensing.

When you complete the self-assessment, please return it to:
Catherine Wittman/Susan Arthur
Children's Council of San Francisco
445 Church St.
San Francisco, CA 94114

If you have any questions or need help completing the self-assessment, feel free to contact us by email at healthyappleaward@gmail.com or by phone at (415) 355-6287. Thank you for your interest and commitment to healthy childhood development.

Self-Assessment of Nutrition, Physical Activity, & Screen Time in Child Care
(For sites serving children age 0 – 5 years old)

Nutrition

*Children begin learning healthy eating habits at a very young age, and their bodies are growing rapidly. Additionally, most children eat 50% to 75% of their daily calories at child care. It is important that food served at child care is nutritious and that providers and staff model and encourage healthy eating habits. **

**Fruits and Vegetables**				
**5.** Offer fresh fruit (not juice) and/or a vegetable (not including French fries, tater tots, hash browns, or dried beans):	☐Rarely or never	☐Sometimes	☐Most of the time	☐Every meal
**6.** Offer fruit that is fresh, frozen, dried or canned in juice (not syrup):	☐Rarely or never	☐Sometimes	☐Most of the time	☐All of the time
**7.** Offer a variety of vegetables, such as dark green, orange, red, and deep yellow vegetables (not including potatoes, corn, and green beans):	☐Less than 1 time per week	☐1-2 times per week	☐2-3 times per day	☐4 or more times per day
**8.** Prepare cooked vegetables without added meat fat, margarine or butter:	☐Rarely or never	☐Sometimes	☐Most of the time	☐All of the time
**Meats, Fats, and Grains**				
**9.** Offer fried or pre-fried potatoes (French fries, tater tots, hash browns):	☐2 or more times per week	☐1 time per week	☐Less than 1 time per week	☐Never
**10.** Offer fried or pre-fried (frozen and breaded) meats (chicken nuggets) or fish (fish sticks):	☐2 or more times per week	☐1 time per week	☐Less than 1 time per week	☐Never
**11.** Offer beans or lean meats (baked or broiled chicken, turkey, or fish):	☐Less than 1 time per week	☐1-2 times per week	☐3-4 times per week	☐1 or more times per day
**12.** Offer sweets or salty foods (pastries, donuts, chips):	☐1 or more times per day	☐One time per week	☐Less than one time per week	☐Never
**13.** Offer high fiber, whole grain foods (brown rice, wheat bread):	☐1 time per week or less	☐2-4 times per week	☐1 time per day	☐2 or more times per day
**Foods Offered Outside of Regular Meals and Snacks**				
**14.** Guidelines provided to parents for food brought in for holidays or celebrations are:	☐Not available	☐Loose guidelines with healthier options included	☐Written guidelines for healthy options, not always enforced	☐Written guidelines for healthier options, usually enforced
**15.** Celebrate holidays with mostly healthy foods and non-food treats:	☐Rarely or never	☐Some of the time	☐Most of the time	☐All of the time

**Beverages**				
**16.** Drinking water is available:	☐Upon request	☐Upon request or during designated water breaks	☐Inside only where it is visible and freely available	☐Inside & outside, it is visible & available for self-serve
**17.** Offer water (not as a substitute for milk when it is a required food component):	☐Never	☐1 time per week	☐2-4 times per week	☐1 time per day or more
**18.** 100% fruit juice is offered:	☐In unlimited portions more than 1 time per day	☐Less than 2 times per day; limited to 4 oz. each occasion	☐No more than 4 oz. per day	☐No more than 4 oz. per day *or never*; parents encouraged to support limit
**19.** Offer sugary drinks (Kool Aid, fruit drinks, sports drinks, sweet tea, soda):	☐More than 1 time per month	☐Less than 1 time per month	☐Rarely, 1-2 times per year	☐Never
**20.** Milk served to children ages 2 – 5 is:	☐Whole or regular	☐2% reduced fat	☐Always, 1-2% low-fat	☐Always 1% or skim/ non-fat
**21.** Flavored milk is served:	☐More than 1 time per month	☐Less than 1 time per month	☐Rarely, 1-2 times per year	☐Never
**Healthy Eating Environment**				
**22.** Meals are served family style:	☐Rarely or never	☐Sometimes	☐Most of the time	☐All of the time
**23.** Providers join children at table for meals; talk informally about trying and enjoying healthy food:	☐Rarely or never	☐Sometimes	☐Most of the time	☐All of the time
**24.** In front of the children, providers consume the same food and drinks as children, and avoid unhealthy foods (e.g., soda, sweets, and fast food):	☐Rarely or never	☐Sometimes	☐Most of the time	☐All of the time
**Feeding Practices**				
**25.** Staff help children decide if they are full before removing plate:	☐Rarely or never	☐Some of the time	☐Most of the time	☐All of the time
**26.** Staff help children decide if they are still hungry before serving additional food:	☐Rarely or never	☐Some of the time	☐Most of the time	☐All of the time
**27.** Staff gently and positively encourage children to try a new or less favorite food:	☐Rarely or never	☐Some of the time	☐Most of the time	☐All of the time
**28.** Food is not used to encourage positive behavior or as punishment:	☐Some of the time	☐All of the time		

Menus and Variety				
**29.** Menus used are:	☐1-week cycle	☐2-week cycle	☐3-week cycle or more without seasonal change	☐3-week cycle or more with seasonal change
**30.** Include a combination of new and familiar foods on weekly menus:	☐Rarely or never	☐Some of the time	☐Most of the time	☐All of the time
**31.** Include foods from a variety of cultures on weekly menus:	☐Rarely or never	☐Some of the time	☐Most of the time	☐All of the time
**Nutrition Education**				
**32.** Offer training on child nutrition (other than food safety and food program guidelines) to staff:	☐Rarely or never	☐Less than 1 time per year	☐1 time per year	☐2 times per year or more
**33.** Provide nutrition education for children:	☐Rarely or never	☐1 time per month	☐2-3 times per month	☐1 time per week
**34.** Offer child nutrition education (workshops, activities and/or take home materials) to parents:	☐Rarely or never	☐Less than 1 time per year	☐1 time per year	☐2 times per year

35. ***Optional:*** *Please add any comments regarding your site's barriers/challenges to meeting the above practices in nutrition, or examples of how your site successfully follows best practices. (Example: CHALLENGE: Parents wish for us to serve their children whole milk at mealtimes. SUCCESS: We invite parents to bring in arts and crafts projects to celebrate children's birthdays instead of sugary treats.)*

Physical Activity

*Regular physical activity has many mental and physical health benefits for children, including reaching and maintaining a healthy weight and reducing the risk of stress and depression. It is recommended that preschool children get at least 120 minutes of varying types of active play time each day, and that staff encourage and model active play. ***

**Active Play and Inactive Time**				
**36.** Active play time (indoor and outdoor) is provided to all children, including those with special needs:	☐45 minutes or less each day	☐46-90 minutes each day	☐91-120 minutes each day	☐More than 120 minutes each day
**37.** Structured (or teacher-led) activities are provided to all children, including those with special needs:	☐1 time per week	☐2-4 times per week	☐1 time per day	☐2 or more times per day
**38.** Outdoor active play time is provided to all children, including those with special needs:	☐1 time per day for 30 minutes or less	☐1 or more times per day for a total of 30-45 minutes	☐1 or more times per day for a total of 45-60 minutes or more	☐2 or more times each day for a total of 60 minutes or more
**39.** During a typical day, excluding naps and meals, children are expected to remain seated:	☐More than 30 minutes at a time, or 15-30 minutes on 3 or more occasions	☐15-30 minutes on 2 occasions	☐15-30 minutes but only 1 occasion	☐No more than 15 minutes at a time
**Play Environment**				
**40.** Portable play equipment (e.g. wheel toys, balls, hoops, ribbons) consists of:	☐Limited variety; children must take turns	☐Some variety; children must take turns	☐Good variety indoors & outdoors; children must take turns	☐Variety indoors & outdoors supporting multiple areas of motor skill development; children may use at the same time
**41.** Outdoor play areas for children including those with special needs, consists of:	☐1-2 different play areas (e.g. sandbox, swing set); no open space for running or track/path for wheeled toys	☐2-3 different play areas; limited space for running and use of wheeled toys	☐Multiple play areas; either an open space for running or a track/path for wheeled toys	☐Multiple areas; open spaces for running, <u>and</u> track/ path for wheeled toys
**42.** Indoor gross motor play area for children, including those with special needs, consists of:	☐Space only suitable for quiet play	☐Space for limited movement (jumping and rolling)	☐Ample space for some active play (jumping, rolling, and skipping)	☐Space for all activities, including running
**43.** A wide variety of fixed play equipment (tunnels, balancing equipment, climbing equipment) that accommodates the needs of all children is:	☐Unavailable at our site	☐Only one type of equipment is available	☐Different equipment is available that suits most children	☐Wide variety of equipment available; suits needs of all children

Provider Behaviors				
**44.** During active play time staff:	☐Supervise play only (mostly sit or stand)	☐Sometimes encourage children to be active	☐Often encourage children to be active	☐Often encourage children to be active; join in active play
**45.** Active play time is:	☐Often withheld for misbehavior	☐Sometimes withheld for misbehavior	☐Never withheld for misbehavior	☐Never withheld for misbehavior; written policy
**Supporting Physical Activity**				
**46.** Support physical activity in classrooms and common areas with:	☐No posters, pictures, or books about physical activity displayed	☐A few posters, pictures, or books about physical activity displayed in a few rooms	☐Posters, pictures, or books about physical activity displayed in most rooms	☐Posters, pictures, or books about physical activity displayed in every room
**Physical Activity Education**				
**47.** Offer staff training in child physical activity (in addition to playground safety):	☐Rarely or never	☐Less than 1 time per year	☐1 time per year	☐2 times per year or more
**48.** Offer parents child physical activity education (e.g., special programs, newsletters, information sheets):	☐Rarely or never	☐Less than 1 time per year	☐1 time per year	☐2 times per year
**49.** Provide physical activity education for children through a standardized curriculum:	☐Rarely or never	☐1 time per month	☐2-3 times per month	☐1 time per week or more

50. ***Optional:*** *Please add any comments regarding your site's barriers/challenges to meeting the above practices in physical activity, or examples of how your site successfully follows best practices (Example: CHALLENGE: We do not have a yard, and the parks nearby are not safe. SUCCESS: Group dance/active time is part of our daily curriculum.)*

Screen Time

*Time spent on the computer, watching TV or videos, and playing video games should be limited in the child care environment. It is recommended that preschool children get no more than 30 minutes of screen time each week. Screen time moderation is a key factor in helping children stay active. ***

Screen Time Provisions				
**51.** The amount of screen time allowed at child care is:	☐More than 2 hours per week	☐2 hours or less per week	☐1 hour or less per week	☐30 minutes per week or less
**52.** Television/DVD viewing includes:	☐All types of programming & videos; limited coordination with curriculum	☐Educational & some commercial programming & videos; some integration with curriculum	☐Mostly educational, age-appropriate programming & videos; many integrated with curriculum	☐Only commercial-free, age-appropriate, educational programming; integrated with the curriculum
**53.** Computers are:	☐Available for use all of the time; time allowed per child is unlimited	☐Available several times during the day; each child is allowed between 30 & 45 min. per day	☐Available only during a set time of day; each child is allowed between 15 and 30 min. per day	☐Available only during a set time of day; each child is limited to 15 min. per day

54. ***Optional:*** *Please add any comments regarding your site's barriers/challenges to meeting the above practices in screen time, or examples of how your site successfully follows best practices. (Examples: CHALLENGE: I am physically unable to lead exercise with the children so I put on a kids' exercise DVD to make sure they're active on rainy days. SUCCESS: My site has no computers, so there is never computer time.)*

Policies		
**Nutrition Policy**		
**55.** Written policy on nutrition and food services is available, followed, and made available to parents upon enrollment:	☐No	☐Yes
**Physical Activity Policy**		
**56.** A policy on physical activity addressing provider behaviors, education and active play opportunities is available:	☐No	☐Yes
**57.** ***Optional:*** *Please add any comments regarding your site's barriers/challenges to meeting the above practices in policies, or examples of how your site successfully follows best practices. (Example: CHALLENGE: I am unsure of what needs to be included in a physical activity policy. SUCCESS: A "sugary drinks" policy for children and staff has helped keep all sugary beverages out of our child care environment.)*		

*Adapted from NAP SACC training materials
** Adapted from Let's Move Child Care websites: http://www.healthykidshealthyfuture.org/activities.html and http://www.healthykidshealthyfuture.org/activities/screentimes.html

THANK YOU for completing this self assessment. One of our staff will follow up with you soon. Please provide your contact information so we can also send you an incentive item for your participation (as supplies last, one per year).

Name:

Address:

City, State, Zip:

Phone Number:

Email Address:

If you have any other questions or comments regarding this self-assessment, feel free to use the space below.
