# Supplementary material for: Healthy apple program to support child care centers to alter nutrition and physical activity practices and improve child weight: a cluster randomized trial
Source: BMC Public Health. 2017 Dec 19;17:965. doi: 10.1186/s12889-017-4951-y (PMC6389251; doi:10.1186/s12889-017-4951-y)
Supplement: Supplementary file 2 — Healthy Apple Award, Excellence in Nutrition, Physical Activity, & Screen Time in Child Care Environments, Goal Setting Worksheet. The CCHP health workers used this document to support child care providers to set goals and develop an action plan for practice improvement. (PDF 119 kb) [file 12889_2017_4951_MOESM2_ESM.pdf]

Healthy Apple Award

Excellence in Nutrition, Physical Activity, & Screen Time in Child Care Environments

Goal Setting Worksheet

Provider Name: ______________________________Date: ____________

Healthy Apple Award Level: *Nutrition*	None	Bronze	Silver	Gold
Healthy Apple Award Level: *Physical Activity*	None	Bronze	Silver	Gold
Healthy Apple Award Level: *Screen Time*	None	Bronze	Silver	Gold
Key Focus Area for Improvement	Nutrition	Physical Activity	Screen Time	
Best Practice for Improvement (1)				
Action Plan				
Date of Completion		Staff Responsible:		
Best Practice for Improvement (2)				
Action Plan				
Date of Completion		Staff Responsible:		
Workshop to Attend				

DRAFT
