# Supplementary material for: Healthy apple program to support child care centers to alter nutrition and physical activity practices and improve child weight: a cluster randomized trial
Source: BMC Public Health. 2017 Dec 19;17:965. doi: 10.1186/s12889-017-4951-y (PMC6389251; doi:10.1186/s12889-017-4951-y)
Supplement: Supplementary file 3 — Healthy Apple Award, Excellence in Nutrition, Physical Activity, & Screen Time in Child Care Environments, Technical Assistance Tip Sheets. Description: The CCHP health workers used and offered this document as a resource for child care providers to plan practice improvements. (PDF 25251 kb) [file 12889_2017_4951_MOESM3_ESM.pdf]

Healthy Apple Award

Excellence in Nutrition, Physical Activity, & Screen Time
in Child Care Environments

Technical Assistance Tip Sheets

Nutrition

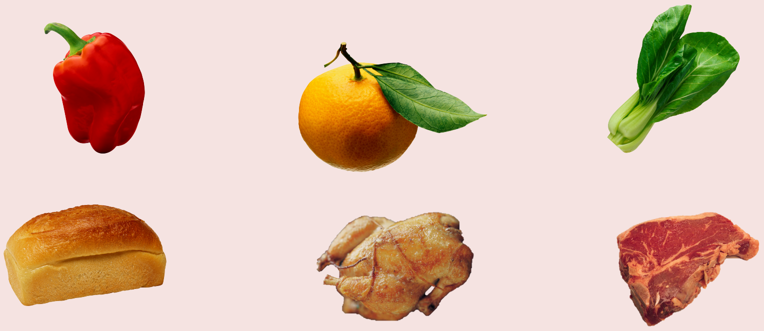

Physical Activity

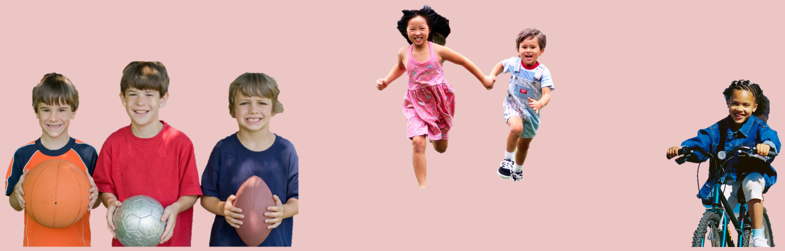

Screen Time

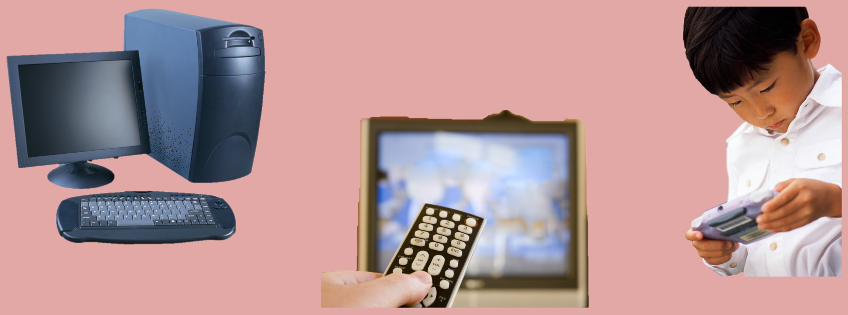

Policies

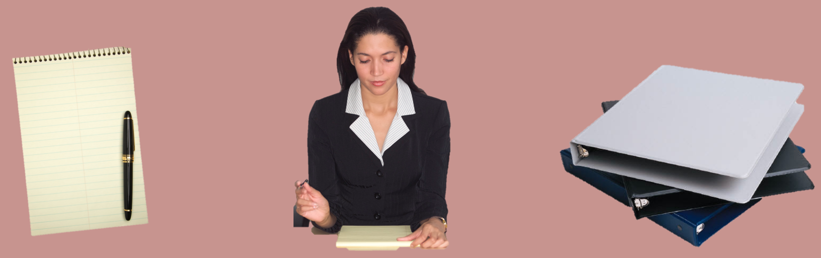

Introduction

On the following pages, you will find several tips to help you to improve upon your current practices in the areas of nutrition, physical activity, and screen time within your child care home or center.

On the left-hand column we have listed the Gold Level best practices, which are the most difficult-to-attain levels of each practice in our Healthy Apple Award Self-Assessment. These practices are listed in the order found within the Self-Assessment, so that you can easily navigate to the item that you are looking for.

The right-hand column contains specific tips and web resources to help you to reach the improvement goals that you have set for yourself.

At the end of each section (Nutrition, Physical Activity, and Screen Time), we have provided several general resources that correspond to that section.

If you have any questions, please contact your Healthy Apple Award consultant or the project coordinator at healthyappleaward@gmail.com or (415) 355-6287.

Thank you!

Please note that this document has been modified from the Nutrition and Physical Activity Self-Assessment for Child Care (NAP SACC) Consultant Technical Assistance Manual, developed by the NAP SACC program, Center for Health Promotion and Disease Prevention, The University of North Carolina, Chapel Hill, NC.

Table of Contents

Nutrition

- Fruits & Vegetables 4
- Meats, Fats, & Grains 5
- Foods Offered Outside of Regular Meals & Snacks 7
- Beverages 8
- Healthy Eating Environment 9
- Feeding Practices 11
- Menus & Variety 12
- Nutrition Education 13

Physical Activity

- Active Play & Inactive Time 15
- Play Environment 16
- Provider Behaviors 18
- Supporting Physical Activity 18
- Physical Activity Education 19

Screen Time

- Screen Time Provisions 21

Policies

- Nutrition Policy 22
- Physical Activity Policy 22

Key Area: Nutrition

Category: Fruits & Vegetables

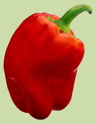 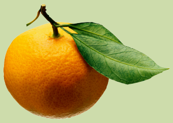 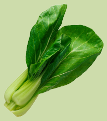

Offer fresh fruit (not juice) and/or a vegetable (not including French fries, tater tots, hash browns, or dried beans) at every meal.	• Buy fresh fruits and vegetables when they are in season or when on sale. Local Farmers Markets are great sources of lower cost fresh produce.<br>• Save preparation-heavy produce for days that require less cooking of other menu items.<br>• Some fresh fruits and vegetables can be purchased already cut up and seeded, ready to eat.<br>• Introduce new vegetables one at a time with familiar favorites to avoid overwhelming children.<br>• Add vegetables into mixed dishes such as casseroles, soups, or sauces; often children won't even notice.<br>• Plant a vegetable garden in the yard or a tomato plant in the classroom and teach children about where food comes from. Research suggests they will be more likely to eat it!<br>• Website for gardening curriculum: http://teamnutrition.usda.gov/Resources/growit.html<br>• Website for community gardens in SF: http://sfrecpark.org/parks-open-spaces/community-gardens-program/request/
Offer fruit that is fresh, frozen, dried or canned in juice (not syrup) all of the time.	• Frozen and canned fruits are a simple alternative to fresh and in some cases may be less expensive. Be sure to purchase fruits canned in juice only and drain before serving.<br>• Choosing fruits packed in their natural juices is helpful in reducing the amount of excess sugar added.<br>• Buy fresh fruits when they are in season or when on sale.

Offer a variety of vegetables, such as dark green, orange, red, and deep yellow vegetables (not including potatoes, corn, and green beans) 4 or more times per day.	• Frozen vegetables are a simple alternative to fresh and in some cases may be less expensive.<br>• Buying vegetables when they are in season or on sale can also help cut costs.<br>• Purchase pre-washed, pre-cut vegetables or vegetables that don't need any preparation such as frozen cut carrots.<br>• Save preparation-heavy produce for days that require less cooking of other menu items.<br>• Canned vegetables are fine to serve some of the time. If the staff takes care in selecting products, excess salt can be minimized. Choose "no salt added" vegetables and low-sodium vegetable soups. Staff can also reduce salt by rinsing the vegetables in water before preparing them.
Prepare cooked vegetables without added meat fat, margarine, or butter	• Vegetables can also be seasoned with herbs, spices, salsa, or lemon juice.<br>• Sauté vegetables with cooking spray or with a tiny amount of canola or vegetable oil.<br>• Mashed potatoes made with chicken broth or low fat buttermilk is great. If serving cheese with vegetables, sprinkle lightly with lower-fat varieties like Parmesan and mozzarella cheese. Serve reduced fat salad dressings, yogurt or salsa with raw vegetables.

Key Area: Nutrition

Category: Meats, Fats, & Grains

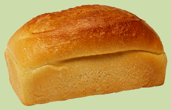
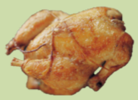
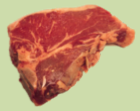

Never offer fried or pre-fried potatoes (French fries, tater tots, hash browns).<br><br>Never offer fried or pre-fried (frozen and breaded) meats (chicken nuggets) or fish (fish sticks).	• For potatoes, instead of frying, bake, mash or add them to soups and stews.<br>• Children accept new foods best when they are offered along with a familiar food. Staff can offer a new food like baked potatoes with pre-fried chicken nuggets.<br>• Children love to dip! Offer baked chicken strips or homemade potato wedges with dipping sauces such as ketchup or BBQ sauce to enhance acceptability.

Offer beans or lean meats (baked or broiled chicken, turkey, or fish) one or more times per day.	• Lower fat versions of high-fat favorites are often available for the same price. Try alternatives like cut-up turkey dogs and deli turkey.<br>• Buying large bags of frozen lean meats such as chicken breasts are cheap alternatives. Separate them into appropriate portions using freezer safe bags or containers before storing so that they do not stick together.<br>• Dried beans are one of the cheapest protein foods available.<br>• Try adding lean ground turkey in place of ground beef to tomato sauce. Beans can also be added to soups, either mashed or whole. Children may not even notice the difference.<br>• Serving new foods alongside items that the children recognize will often increase their acceptance. For example, serve a grilled chicken patty on a bun with the same side items you would normally serve with a hamburger.
Never offer sweets or salty foods.	• Staff can offer alternative snack foods that have less sugar and/or salt, such as pretzels and low sodium crackers. Some items, such as ginger snaps and graham crackers, are lower in sugar and are popular with children.<br>• Many wholesome and healthy foods such as fruit and grains contain natural sugars. Children can get all the nutrients they need, including sugars, without consuming foods with a lot of added sugar, like cookies and muffins.
Offer high fiber, whole grain foods 2 or more times per day.	• It may be a challenge to introduce whole grain alternatives to children who are used to eating only enriched flour grains. Staff can introduce these foods gradually, by mixing half white pasta with half whole-wheat pasta, or substituting the whole grain version of favorite crackers.<br>• Some foods that children already love have whole grains and are high in fiber. Staff can serve Cheerios® or oatmeal instead of sugary cereals with low nutrient value.

Key Area: Nutrition

Category: Foods Offered Outside of Regular Meals & Snacks

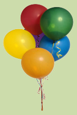

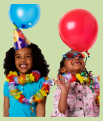

Guidelines provided to parents for food brought in for holidays or celebrations are written and usually enforced.	• Give parents ideas for quick and nutritious foods that can be easily purchased and are suitable for birthdays and other celebrations (ex. already sliced fruit or vegetable trays, mini muffins, etc.).<br>• Encourage non-food treats, like bubbles and party hats.<br>• Encourage parents to think about their child's favorite things, other than foods, that can be included in the celebration. This might include a favorite song, book, or game. Offer to include these special things in planning activities for a child's birthday. Often the biggest treat of all is for the parent to make a brief visit to school to help celebrate the birthday.<br>• Sample of food guidelines for parents: http://www.peachtreepresbyterianpreschool.org/ProgramsAndResources/PPCPreschoolCelebrationFoodGuidelines.pdf
Always celebrate holidays with mostly healthy foods and non-food treats.	• As part of a celebration, fill a piñata with toothbrushes, raisins, stickers, jump ropes, and other fun foods and toys.<br>• Staff should inform parents early of their policy to serve healthier foods for holidays. It's easier to get buy-in for this when parents enroll their children and learn that the facility is working hard to create the healthiest environment possible for their children.<br>• Use this as an opportunity to investigate other cultures and their traditions.<br>• Tips for easy, healthy celebrations: http://www.fns.usda.gov/eatsmartplayhardhealthylifestyle/quickandeasy/celebrations.pdf

Key Area: Nutrition<br>Category: Beverages	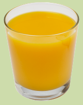 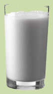 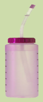
Drinking water is available inside & outside. It is available for self-serve.	• Find creative ways to make water easily accessible to children. Try child-height water coolers or keep pitchers of ice water and small paper cups in classrooms.<br>• Make drinking water fun for children by adding lemon slices before serving it or providing straws for children to sip water from a cup.<br>• Avoid unwanted spills by filling pitchers of water only part way, by using pitchers with lids, and by helping younger children when pouring water.<br>• Simple flavored water recipes: http://www.examiner.com/article/4-easy-flavored-water-recipes
Offer water (not as a substitute for milk when it is a required food component) one time per day or more.	• Water may be offered between meals to quench thirst, immediately following a meal, or when an individual child drinks his/her milk and is still thirsty.<br>• Offer water whenever a child is thirsty, and at fixed times of day (such as mid-morning and pm snack).
No more than 4 ounces of 100% juice is offered per day. Parents are encouraged to support this limit.	• Juice should be limited to days when high fat foods are not consumed, since juice suppresses fat burning, unlike water.<br>• Help staff identify easy to eat alternatives to juice like orange sections, fruit salad, or banana halves.<br>• If a child has already had juice with a meal or snack, staff should offer water instead.<br>• Juice provides excess calories, sugar and acid, and increases risk of obesity, type II diabetes, and cavities for children.

Never offer sugary drinks (Kool Aid, fruit drinks, sports drinks, sweet tea, soda).	• Provide information to parents on the amount of sugar in soft drinks and other beverages and its effect on children's health. Develop a beverage policy and provide parents with a list of healthy alternatives.<br>• Resources for parent education about sugary drinks: http://www.sfgov3.org/index.aspx?page=1872
Milk served is always 1% or skim/non-fat.<br><br>Flavored milk is never served.	• Before making a practice change, send information home for parents with reassurance that skim and low- fat milk are safe and healthy choices for young children. Parents can also be encouraged to make the switch at home.<br>• Make the milk switch without much fuss. Milk taste-tests done with children show that most cannot distinguish differences without prompting from an adult. Most children will accept whatever type of milk is poured in their glass and never know the difference.<br>• Make the switch gradually by going from whole to 2% to 1% to skim over time.
<u>Key Area:</u> Nutrition<br><br><u>Category:</u> Healthy Eating Environment	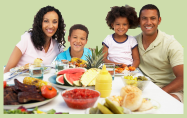
Meals are always served family style.	• Use 1⁄2 pints of milk so that pouring is made easier for children. Smaller pitchers and serving bowls also minimize the likelihood of spills.<br>• Ask children to help with clean up after a spill, taking care to help them feel good about helping vs. feeling bad about the spill.<br>• Assigning children jobs like setting the table, bringing food to the table, and clearing and cleaning the table after eating can actually make the staff's job easier. After the children get the hang of their duties, meals will run much smoother.<br>• Provide each item in several small bowls so if a child touches the food directly it can be discarded with limited waste.

Providers join children at table for meals and talk informally about trying and enjoying healthy food.	• Help create schedules that allow staff to have their break during naptime, not mealtime. This will free up staff and enable them to eat with the children.<br>• Inform staff when they are hired that they are expected to be good role models. They can't expect a child to try a new food if they won't try it themselves. Include this in your staff policy.<br>• Involve staff with taste-testing new menu items to get their buy-in.
In front of the children, providers consume the same food and drinks as children, and avoid unhealthy foods (e.g., soda, sweets, and fast food).	• Ask the staff to take and consume a small amount of everything served and avoid negative comments in front of the children about foods they don't like.<br>• Give staff short "coffee" breaks outside of the classroom and ask them to consume only water or the beverages served to children during meal and snack times in the classroom.<br>• Make these expectations clear to staff when they are hired or present facility policy changes at staff meetings and ask for everyone's cooperation.<br>• Work with staff to understand that role modeling healthy eating is much like role modeling other things like polite communication (saying please and thank you, not interrupting others when talking) or speaking in a volume appropriate to the circumstances – quieter inside vs. outside. Most staff would not think twice about the importance of modeling these behaviors.

Key Area: Nutrition

Category: Feeding Practices

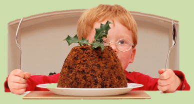
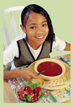
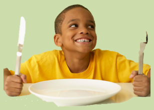

Staff help children decide if they are full before removing the plate.<br><br>Staff help children decide if they are still hungry before serving additional food.	• Work with the staff to remind them that a child's eating behavior may vary day to day. Avoid waste by serving small portions to begin with.<br>• Let staff know that it's normal for children not to eat everything on their plates.<br>• Ask staff to apply the approach of asking whether they are full with all children. Another strategy is to limit offers of seconds to everyone to only those foods of lower caloric value such as fruits and vegetables.
Staff gently and positively encourage children to try a new or less favorite food.	• Introduce new foods one at a time. Offer a very small amount at first; children will learn new flavors and textures without being overwhelmed.<br>• Repeat exposure. It can take 7 attempts before a child will taste a new food.<br>• Introduce new foods in whole form at circle time. Allow them to touch, smell, and taste at this time. Graph observations.<br>• Offer new foods at the beginning of the meal when children are hungry and more likely to try something new.<br>• Enlist the help of an eager child who is usually open toward trying new foods. Children will be more willing to try a new food if another child has already tried it and liked it.<br>• Encourage, but don't force a child to try a new food. Children may be more willing to try a new food when there is no pressure and they are in control.<br>• Tell parents about new foods their child may have tried at the facility and encourage them to try these same foods at home.
Food is not used to encourage positive behavior or as punishment.	• Reward children with non-food options. Some suggestions are praise, line leader, teacher's assistant, or stickers.

Key Area: Nutrition<br>Category: Menus and Variety	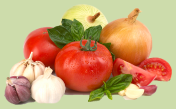 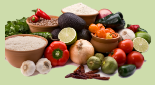 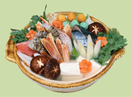
Menus are on a 3-week cycle or more with seasonal change.	• Send a food service staff person to a local CACFP training for menu planning assistance. Offer to obtain and provide free meal planning resources from the USDA and Team Nutrition to the facility director or cooks.<br>• Variety doesn't have to be expensive. Consider frozen meats and vegetables that can be purchased in bulk and used as needed.<br>• Using seasonal items, such as apples in the fall, can reduce costs.<br>• How to plan cycle menus: http://www.nfsmi.org/documentlibraryfiles/PDF/20100916051200.pdf<br>• In-season produce guide: http://www.pcfma.com/pdf/Eat_in_season.pdf
Include a combination of new and familiar foods on weekly menus.	• Add a few new foods to each cycle so that children have repeated exposure to new foods.<br>• Introduce only one new food at any one meal. Try to serve at least one known favorite at each meal so no one goes hungry.<br>• Introduce new foods through a fun activity with children such as a tasting party or work them into the theme of the week.<br>• Introducing new items could simply mean replacing an ingredient while still using the same recipe. Try using ground turkey instead of beef in spaghetti, substitute green beans for peas, or substitute brown rice for white rice. Help staff organize menus so that easy, no prep snacks are combined with harder to prep meals.

Include foods from a variety of cultures on weekly menus	• Encourage staff to get involved by sponsoring an ethnic recipe contest.<br>• Include foods of ethnic significance to staff members.<br>• Choose a country or ethnicity and have a celebration including regional food, music, and fun activities.<br>• Facilities can prepare some of their own cultural foods with minimal effort and time. Many regional foods are readily available canned or frozen. Look for easy ways to reduce calories and fat such as, using low fat cheeses, chicken or turkey instead of beef, etc.<br>• Recipes from all around the world: http://www.circletimekids.com/recipes/
Key Area: Nutrition<br>Category: Nutrition Education	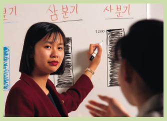 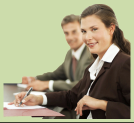
Offer training on nutrition (other than food safety and food program guidelines) to staff 2 times or more per year.	• There are many community agencies, such as Cooperative Extension Services, that can help provide nutrition education training to staff. The CACFP also provides nutrition trainings to child care facilities.<br>• Provide staff with individual training plans that include some nutrition education. Schedule trainings on a variety of topics each year and consider integrating nutrition into trainings that address other issues (i.e. behavior).<br>• Free online nutrition training: http://infanttoddler.tamu.edu/courses/courseListByCatID.php?catid=15
Provide nutrition education for children one time or more per week.	• Suggest nutrition education materials designed for young children (e.g. Color Me Healthy, Chef Combo, Healthy Start).<br>• Suggest ways to integrate nutrition education with other learning activities such as numbers, counting or sorting. For example, children can count how many fruits and vegetables they eat at a meal, or sort foods into groups of the same color.

Offer training on child nutrition (workshops, activities, and/or take home materials) to parents 2 times or more per year.	• Suggest that facilities send children home with nutrition education materials, such as the NAP SACC parent handouts. The Color Me Healthy program also provides newsletters for parents with nutrition information.<br>• Offer simple ways to integrate nutrition issues and education into parent meetings such as announcing a facility's involvement in the Healthy Apple program and announcing other efforts to promote healthy foods and healthy weight.<br>• There are community resources available to help provide nutrition education to parents. Resources may include: Cooperative Extension, CACFP administrators, and dietitians in health departments. Facilities may identify parents or volunteers with training in nutrition or who work in settings with dietitians.

Nutrition Resources

- My Plate for Preschoolers: Healthy meal ideas; great for providers to share with parents
    - http://www.choosemyplate.gov/preschoolers.html
- Healthy Habits for Life: Downloadable unit plans for nutrition, starring Sesame Street characters
    - http://kidshealth.org/classroom/index.jsp?Grade=cc&Section=hhfl
- Let's Move! Child Care: Tips & resources to help providers meet nutrition goals
    - http://www.healthykidshealthyfuture.org/welcome.html
- Charles B. Wang Community Health Center: Downloadable, printable handouts in English and Chinese for providers to share with parents
    - http://www.cbwchc.org/project/brochures/brochures_ped.asp#child
- Links to Healthy Recipes – CDC: Links to sites with healthy recipes – good for providers and parents
    - http://www.cdc.gov/healthyweight/healthy_eating/recipes.html
- Grow It, Try It, Like It! – USDA: Tools for providers to get a garden started, along with preschool-friendly nutrition curriculum centered on foods that they grow.
    - http://teamnutrition.usda.gov/Resources/growit.html
- Cooperative Extension San Mateo/San Francisco Counties: USDA-funded educational programs about healthy eating, price-conscious food choices, and food safety. They offer a series for parents and for educators.
    - http://cesanmateo.ucanr.edu/Nutrition,_Family_and_Consumer_Science/

Key Area: Physical Activity

Category: Play and Inactive Time

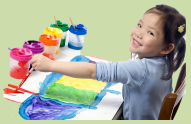
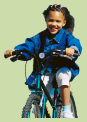
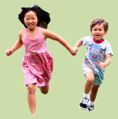

Active play time (indoor and outdoor) is provided to all children, including those with special needs more than 120 minutes each day.	• Review the daily schedule to see where and how active time can be added. If there is no schedule, encourage them to create one.<br>• The 120 minutes of physical activity time doesn't have to be obtained all at once. Include 2-3 blocks of active playtime across the day both inside and outside.<br>• Provide information to parents about the importance of physical activity for children's physical, mental and emotional health.<br>• Incorporate active learning into daily lesson plans; allowing children to learn through movement.<br>• Encourage the facility to use credible physical activity curricula (i.e. Be Active Kids), which includes a number of fun activities incorporating learning and active playtime.
Structured (or teacher-led) activities are provided to all children, including those with special needs, 2 or more times per day.	• Staff can add activities into their daily lesson plans. Many appropriate lesson plans and activities are available on the Internet or in books (see resources).<br>• Hold a training session for staff to help staff become more comfortable and teach staff age appropriate activities.
Outdoor active play time is provided to all children, including those with special needs one or more times per day for a total of 45-60 minutes or more.	• Work with the facility to redefine their outdoor space. If outdoor space is being used for adjacent parking, work with the facility to relocate cars to neighborhood areas and reclaim this area. Sand and mulch will have to replace the hard surface.<br>• Encourage the facility to consider alternative spaces for active play, such as neighboring elementary schools or parks.<br>• Map of parks in SF: http://www.sfparksalliance.org/visit/find-events-parks

<table>
<tr>
<td>During a typical day, excluding naps and meals, children are expected to remain seated 15-30 minutes, only on one occasion.</td>
<td>

Set reasonable expectations for children’s behavior while active. Setting expectations will help with class control. Use hand or verbal signals to signal children to maintain control.
Color Me Healthy and Be Active Kids are great resources for improving this practice. For example, the Color Me Healthy curriculum has lessons that incorporate physical activity into circle time with imaginary trips, music and fun!
Go over the daily schedule with the facility staff and discuss the sedentary periods. Make sure long transitions are removed or shortened with added activities.

</td>
</tr>
<tr>
<td colspan="2"><u>Key Area:</u> Physical Activity
<u>Category:</u> Play Environment
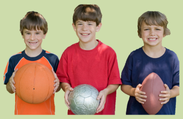 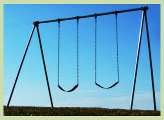 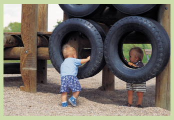</td>
</tr>
<tr>
<td>Portable play equipment (e.g. wheel toys, balls, hoops, ribbons) consists of lots of variety indoors and outdoors. Children may use equipment at the same time.</td>
<td>

Encourage the facility to create a budget to buy or replace portable equipment once or twice a year. Inform directors of discount catalogues or websites such as Discount School Supply or Teacher Storehouse. Ask parents to donate portable equipment such as wheeled toys, balls, jump ropes, etc.
Encourage staff to inventory portable play equipment, looking at the variety available. Recommend equipment such as: balls of varied sizes and material (from rubber balls that are very large and can only be pushed around, to smaller foam ones that can be held in one hand and thrown), hoops (they can be used to roll, toss, or “hula”), wheeled toys, and wagons. Another piece of portable play equipment that is popular is a parachute, which is a great teaching tool as well.
Help the facility review its daily schedule. Fewer children at one time in the play space will encourage sufficient use of equipment by most children.
Have a clean, kid friendly space that allows children to freely access all portable equipment and easily put it back in its place.

</td>
</tr>
</table>

Outdoor play areas for children, including those with special needs, consist of multiple areas. There are open spaces for running and there is a track or path for wheeled toys.	• Encourage the director to apply for a grant or budget for a bike track or path to help enhance the outdoor environment.<br>• A path doesn't have to be fancy. A flat dirt area that runs through the mulch or grass will work fine.<br>• Take a look at the layout of play equipment. Perhaps there is too much for a small space. Think about removing items that may allow for more running space.<br>• Potential grant resources: http://www.buildingchildcare.net/uploads/pdfs/Potential-Grant-Resources-List.pdf
Indoor gross motor play area for children, including those with special needs, consists of space for all activities, including running.	• Encourage the facility to examine the space they do have; are there large rooms that aren't being used? Can classrooms be reorganized (moving bookcases or tables) to allow whole body movement?<br>• Encourage the facility to examine their play schedules to allow for fewer children to be in the available indoor play space at a time, giving more space for children to move about.
A wide variety of fixed play equipment (tunnels, balancing equipment, climbing equipment) that accommodates the needs of all children is available.	• Encourage the use of recycled materials, such as old tires, for fixed play equipment. Tires can be arranged for children to run in, out and around, as well as to swing, climb and jump off of (12"-18" off the ground).<br>• Consider "re-designing" the playground's layout using natural barriers such as flowerbeds, an herb garden, or trees. This will provide different areas for the children and will present natural "tracks" around which children can run or ride on wheeled toys. Playground "screens" can be made using posts and fabric to create a divider that children can push balls under, throw balls over, or run around.<br>• Review the requirements for playground areas, addressing issues of surfacing under play equipment (9-12" of chips, mulch, or sand needed); fall zones (6 feet needed), and equipment height (should be 4 feet or less).

Key Area: Physical Activity

Category: Provider Behaviors

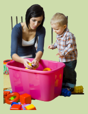
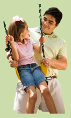
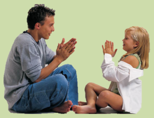

During active play time, staff often encourage children to be active and join in play.	• Staff can support children's physical activity by asking questions ("how does it feel to ride your Big Wheel around the play ground?); by offering encouragement ("Way to go! That was a great jump!"); or just through smiles and laughter during their active time.<br>• Remind staff that they don't have to be experts to model physical activity and to encourage children's activity.<br>• Encourage the facility to promote physical activity among its staff. Establish a facility-wide "competition" among staff using pedometers (step counters). Whoever can walk the most steps at the child care facility over a week period wins a prize. Also, remind the director to be a role model when in the classroom.
Active play time is never withheld for misbehavior. There is a written policy about this.	• Encourage the facility to develop strategies to encourage good behavior through positive reinforcements or use some type of "time out" period, which is not associated with loss of active playtime.<br>• Work with parents to help them understand the importance of physical activity for young children. Parents can also help develop a discipline model that can be used both at school and home.

Key Area: Physical Activity

Category: Supporting Physical Activity

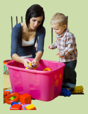
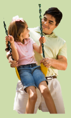
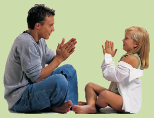

Support physical activity in classrooms and common areas with posters, pictures, or books about physical activity displayed in every room.	• You can take pictures of children being active or have children create their own pictures of them being active with their families and friends – hang them on the wall.<br>• Lots of child care facilities work with their local library branch to borrow books on a biweekly basis. This can be a free resource for all classrooms to take advantage of and can allow children to read actions books that require them to move.

Key Area: Physical Activity

Category: Physical Activity Education

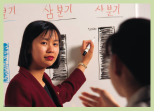

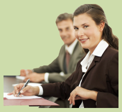

Offer staff training in physical activity (in addition to playground safety) 2 times per year or more.	• Explain to the staff that these types of trainings are important for teaching child development. Encourage all staff, including the facility directors, to participate in such trainings, because it helps keep staff informed about age-appropriate activities and how to successfully implement curricula.<br>• Suggest that the facility look to other organizations to provide training on-site or at the organization. Such organizations include Cooperative Extension Services, local health departments, community colleges and universities, the public school system or voluntary health associations.<br>• Work with the facility directors to examine opportunities to integrate physical activity training within other in- service training. Suggest the facility close one day each year and provide a required day of training for the staff.
Offer parents physical activity education (e.g. special programs, newsletters, information sheets) 2 times per year.	• Suggest that the staff include a discussion of the importance of physical activity and opportunities for physical activity at the facility as the parent is considering placing their child there and at parent orientation. Informal discussions can occur as parents drop off or pick up their children. Suggest brief discussions and announcements that focus on physical activity and obesity prevention at parent meetings.<br>• Suggest that the facility include brief articles in newsletters about fun physical activity opportunities going on at the facility and include comments from children about their favorite ways to be active. Posting information on a bulletin board may also be helpful.

Provide physical activity education for children through a standardized curriculum one or more times per week.	• Explore with and advise the director and staff about opportunities for integrating physical activity into current lessons, which do not require significant schedule changes.<br>• Offer examples of other facilities' schedules and ways they promote a variety of physical activities.<br>• Inform staff about upcoming trainings in Be Active Kids or other physical activity curricula.<br>• Provide information to parents about the importance of physical activity for physical, social and mental health.<br>• Invite parents to visit school and participate in organized physical activity sessions.

Physical Activity Resources

- My Plate for Preschoolers: Outdoor physical activity ideas; great for providers to share with parents
  - http://www.choosemyplate.gov/preschoolers.html
- Healthy Habits for Life: Downloadable unit plans for physical activity, starring Sesame Street characters
  - http://kidshealth.org/classroom/index.jsp?Grade=cc&Section=hhfl
- Let's Move! Child Care: Tips & resources to help providers meet physical activity goals
  - http://www.healthykidshealthyfuture.org/welcome.html
- Charles B. Wang Community Health Center: Downloadable, printable handouts in English and Chinese for providers to share with parents
  - http://www.cbwchc.org/project/brochures/brochures_ped.asp#child
- Parks in San Francisco: Online tool to find parks & recreation areas in specific neighborhoods; clicking an icon on the map brings up a photo and description of the park
  - http://sfrecpark.org/ParksMapPage.aspx#/?i=2
- P.E. Central: Ideas for structured physical activity
  - http://www.pecentral.org/lessonideas/searchresults.asp?category=50
- San Francisco Public Library: Find the nearest branch and visit it to borrow books about physical activity.
  - http://sfpl.org

Key Area: Screen Time

Category: Screen Time Provisions

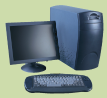 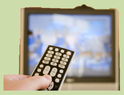 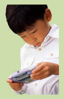

The amount of screen time allowed at child care is 30 minutes per week or less.	• Schedule each day to be full of non-screen time activities. There will be less downtime to fill.<br>• Allow children to help with daily routines like setting up/cleaning up meals or craft projects. Try to make these routines enjoyable by integrating song and dance.<br>• Keep high-interest games and learning toys available for use during times when no activities are scheduled.<br>• Ask parents how they reduce screen time at home.<br>• Create a written policy regarding screen time that can be shared with parents.
Television/DVD viewing includes only commercial-free, age-appropriate, educational programming. It is integrated with the curriculum.	• Choose educational programs that complement your existing curriculum. Plan meaningful learning activities that enhance what children learned from the show.
Computers are available only during a set time each day. Each child is limited to 15 minutes per day.	• Use a timer for children using the computer or playing video games.

Screen Time Resources

- Let's Move! Child Care: Tips & resources to help providers meet screen time goals
  - http://www.healthykidshealthyfuture.org/welcome.html
- Charles B. Wang Community Health Center: Downloadable, printable handouts in English and Chinese for providers to share with parents
  - http://www.cbwchc.org/project/brochures/brochures_ped.asp#child
- Mayo Clinic: Basic tips for reducing screen time; good for providers to share with parents
  - http://www.mayoclinic.com/health/children-and-tv/MY00522/NSECTIONGROUP=2
- Screen Time Reduction Strategies for Early Childhood Professionals – University of Washington: Strategies for providers at their sites and some to share with parents; includes sample screen time policy
  - http://depts.washington.edu/tvhealth/resources_prof.htm

Key Area: Policies

Category: Nutrition Policy

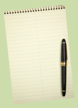
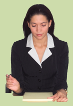
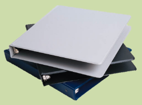

Written policy on nutrition and food services is available, followed, and made available to parents upon enrollment.

- Suggest that facilities use the model policies included in the NAP SACC toolkit as a guide for creating their own nutrition policy.
- Suggest that facilities discuss and document existing nutrition practices and incorporate those into a healthy food policy.
- Use scheduled staff in-service time to discuss nutrition issues and develop a healthy nutrition policy.
- Sample policies are found at the end of the PDF: http://health.mo.gov/living/wellness/nutrition/eatsmartguidelines/pdf/Model_Policies.pdf

Key Area: Policies

Category: Physical Activity Policy

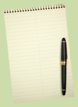
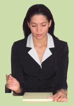
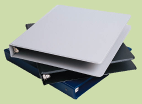

A policy on physical activity addressing provider behaviors, education, and active play opportunities is available.

- Suggest that the facility use the model policies included in the NAP SACC toolkit as a guide for creating their own physical activity policy.
- Work with staff to identify the physical activity-related practices that are currently in place. Explore with staff how to build on existing strengths and create an environment that regularly promotes fun and varied physical activity opportunities.
- Advise how staff, teachers, parents and community partners can all provide invaluable input in developing a policy. Share examples from others that involve parents.
- Sample policies are found at the end of the PDF: http://health.mo.gov/living/wellness/nutrition/eatsmartguidelines/pdf/Model_Policies.pdf
