# Supplementary material for: Healthy apple program to support child care centers to alter nutrition and physical activity practices and improve child weight: a cluster randomized trial
Source: BMC Public Health. 2017 Dec 19;17:965. doi: 10.1186/s12889-017-4951-y (PMC6389251; doi:10.1186/s12889-017-4951-y)
Supplement: Supplementary file 4 — Title: Healthy Apple Award Consultant Follow Up Instructions, June 4, 2013. Description: CCHP health workers followed the instructions detailed in this document to provide one-on-one support to child care providers after they completed the initial HAP self-assessment. (PDF 65 kb) [file 12889_2017_4951_MOESM4_ESM.pdf]

Healthy Apple Award Consultant Follow Up Instructions, June 4, 2013

Steps *prior* to visit:

1. Email or snail mail providers notifying them that you will be calling them to set up a Follow-Up meeting and to request they review the Self-Assessment. Print (even if e-mailing because you may want to have it handy at the Follow-Up meeting) and attach the PDF of their completed Self-Assessment to the email or letter. See my email of 5.29 for email/letter copy suggestions and for the PDFs.

2. Call to set up Follow-Up meeting *AND* to ask them questions using the form: "Pre-Follow-Up Call with Provider to Conduct Phone Questionnaire and Set Up Visit." (You got copies of it at our 5.29 meeting). This phone call may take some time so plan accordingly. If the provider is short on time at least ask the 4 **bolded/*** ** questions (numbers: 1, 4, 6 & 8).

3. To prep for your meetings, review the "Consultant Guide forms" you received for each provider at our 5.29 meeting. Assess the level of easy/difficulty for making changes and consider what you think they may want to focus on. Read the following documents being mindful to customize for the needs of each provider:

   - "How to Use Consultant Guide" sheet
   - Review the "Technical Assistance Tip Sheets" in your Healthy Apple binder (behind the purple divider sheet).
   - See if any Appendix items (also in the HAA binder, after the yellow divider sheet) may be useful to bring with you to the meeting. Make copies as needed.

Review Child Care Wellness Collaborative (CCWC) web site's online resources to see if any are appropriate for you to refer providers to. The URL is: https://sites.google.com/site/childcarewellnesscollaborative/online-assessment. Click on these links for specific topic links:

General Children's Health   Nutrition   Physical Activity   Screen Time

Steps *During* Visit:

4. At Follow-Up Meeting be sure to bring the following documents:
   A. Pre-Follow Up Call/Questionnaire Sheet if you didn't ask all questions on the call.
   B. Consultant Guide
   C. Goal Setting Worksheet/Indoor & Outdoor Observation sheet
   D. HAA Binder for you to consult
   E. Copy of Self-Assessment PDF in case they lost theirs or didn't print it.
   F. Any copies of Tip Sheet Appendix handouts that you want to give to provider.

5. Give overview of HAA. Discuss how they ranked. Discuss area they'd like to focus on and what they think is feasible and realistic for them to address and

improve upon before September. Determine goals, create action plan and write down on Goal Setting Worksheet. Tell them you will mail them a copy of the GSW. Also, encourage them to take the Self-Assessment again before Sept. 15.

6. Give any handouts and resources to assist them in achieving goals. (You will write these down on the GSW, too.)
7. Record Indoor and Outdoor Observations (use sheet on back of Goal Setting Worksheet to record).

Steps *After* Visit:

8. Return to office. Review Goal Setting Worksheet. Determine additional resources you want to offer provider (eg. hard copies of Technical Assistance Tip Sheets, Appendix items and/or links to online CCWC resources at: https://sites.google.com/site/childcarewellnesscollaborative/online-assessment.

General Children's Health **Nutrition** **Physical Activity** **Screen Time**

Write additional resources offered on Goal Setting Worksheet.

9. Make copies of 3 forms as listed below and give one of each form to Catherine Wittman or Susan Arthur:
   1. * Goal Setting Worksheet (2 copies)
   2. * Observation Sheet (1 copy)
   3. Pre-Follow Up Call/Phone Questionnaire Sheet (1 copy)
   - * You also have the option of recording the GSW & Observation sheet data online at: http://www.surveymonkey.com/s/PHTQ92G

   4. Copy any Tip Sheets you want to send to provider.

10. Mail 2nd copy of GSW sheet to provider and insert any hard copy handouts like Tipsheets.

11. Keep original forms for your own records:

    1. GSW worksheet
    2. Observation sheet
    3. Pre-Follow Up Questionnaire

12. Rinse, repeat. (That's a joke ☺)!

Let us know if you have any questions. Thank you so much!

Susan and Catherine
