# Supplementary material for: Healthy apple program to support child care centers to alter nutrition and physical activity practices and improve child weight: a cluster randomized trial
Source: BMC Public Health. 2017 Dec 19;17:965. doi: 10.1186/s12889-017-4951-y (PMC6389251; doi:10.1186/s12889-017-4951-y)
Supplement: Supplementary file 5 — Example Healthy Apple Program Consultant Guide. Description: The CCHP health workers used this template to summarize the target best practices and action plans that child care providers selected. (PDF 454 kb) [file 12889_2017_4951_MOESM5_ESM.pdf]

Example Healthy Apple Program Consultant Guide

Consultant:
Provider Name:
Facility Name:
Type of Child Care: *Center*
Number of Children Enrolled:

Current Award Levels: N – *None* PA – *None* ST – *Gold*

Target Best Practice	Current Score & Description	Next Step Up	Tip Sheet Page #
Offer beans or lean meats (baked or broiled chicken, turkey, or fish): *1 or more times per day.*	(1) Less than 1 time per week	(2) 1-2 times per week	5
Structured (or teacher-led) activities are provided to all children, including those with special needs: *2 or more times per day*	(1) 1 time per week	(2) 2-4 times per week	14
Offer staff training in child physical activity (in addition to playground safety): *2 times per year or more*	(1) Rarely or never	(2) Less than 1 time per year	18
Offer parents child physical activity education (e.g., special programs, newsletters, information sheets): *2 times per year*	(1) Rarely or never	(2) Less than 1 time per year	18
